# Association between intrapartum fetal pulse oximetry and adverse perinatal and long‐term outcomes: A systematic review and meta‐analysis

**DOI:** 10.1002/ijgo.70242

**Published:** 2025-06-09

**Authors:** Jill M. Mitchell, Siobhan Walsh, Laura J. O'Byrne, Virginia Conrick, Ray Burke, Ali S. Khashan, John R. Higgins, Richard A. Greene, Gillian M. Maher, Fergus P. McCarthy

**Affiliations:** ^1^ Department of Obstetrics and Gynaecology University College Cork Cork Ireland; ^2^ INFANT Research Centre Cork Ireland; ^3^ UCC Library University College Cork Cork Ireland; ^4^ Tyndall National Institute Cork Ireland; ^5^ School of Public Health University College Cork Cork Ireland; ^6^ National Perinatal Epidemiology Centre University College Cork Cork Ireland

**Keywords:** blood gas monitoring, fetal heart rate monitoring, fetal monitoring, fetal oxygen saturation, FSpo_2_, intrapartum, labor, oximetry

## Abstract

**Background:**

Fetal pulse oximetry may improve intrapartum fetal evaluation by providing a non‐invasive measurement of fetal oxygen saturation (FSpo_2_).

**Objectives:**

To assess the association between abnormal intrapartum FSpo_2_ and perinatal and long‐term neurodevelopmental outcomes, and to evaluate if the addition of the measurement of FSpo_2_ to established forms of fetal monitoring, such as fetal heart rate monitoring, affects birth, perinatal, and long‐term neurodevelopmental outcomes.

**Search Strategy:**

We conducted a comprehensive search of PubMed, EMBASE, CINAHL, The Cochrane Library, Web of Science, ClinicalTrials.gov, and WHO ICTRP from database inception through February 2024, with no restrictions on date, geographic region, country income level, or language.

**Selection Criteria:**

Studies involving women in labor with a cephalic baby were included. Two interventions were reviewed: (1) low FSpo_2_ (<30%), and (2) the use of fetal pulse oximetry during labor.

**Data Collection and Analysis:**

Independent reviewers screened studies, extracted data, and assessed quality using the Risk of Bias tool and the Newcastle‐Ottawa Scale. The approach evaluated evidence certainty. A random‐effects meta‐analysis followed PRISMA and MOOSE guidelines.

**Main Results:**

Forty‐seven studies with 13 071 mother‐infant pairs were included. FSpo_2_ <30% was associated with umbilical artery pH <7.15 (odds ratio [OR] 7.86, 95% confidence interval [CI] 3.29–18.75, *I* ^2^ = 71%, *P* < 0.001), 5‐min Apgar score less than 7 (OR 16.63, 95% CI 5.64–49.01, *I* ^2^ = 30%, *P* < 0.001) and NICU admission (OR 5.89, 95% CI 1.73–20.01, *I* ^2^ = 0%, *P* < 0.005). FSpo_2_ monitoring combined with fetal heart rate monitoring was associated with lower odds of cesarean section for non‐reassuring fetal status (OR 0.59, 95% CI 0.40–0.86, *I* ^2^ = 71%, *P* = 0.006) without impacting 5‐min Apgar scores <7 (OR 0.66, 95% CI 0.37–1.17, *I* ^2^ = 0%, *P* = 0.160) or neonatal intensive care unit admissions (OR 0.98, 95% CI 0.82–1.18, *I* ^2^ = 0%, *P* = 0.840).

**Conclusion:**

FSpo_2_ monitoring combined with fetal heart rate monitoring may reduce unnecessary cesarean sections for suspected fetal distress without affecting short‐term neonatal outcomes. The association between FSpo_2_ <30% and adverse perinatal outcomes supports its potential as a valuable adjunct in intrapartum monitoring.

## 1 INTRODUCTION

Intrapartum fetal monitoring aims to improve perinatal outcomes while avoiding unnecessary operative interventions.^1^ Current international guidelines endorse intermittent auscultation of the fetal heart rate (FHR), supplemented by continuous cardiotocography (CTG), as the reference standard for identifying potential fetal deterioration and to allow for timely and effective intervention to prevent adverse outcomes secondary to fetal hypoxia.^2^, ^3^, ^4^, ^5^ Despite its status as the established benchmark in care, there is a pervasive consensus that current fetal monitoring devices based on heart rate do not prevent or accurately detect fetal hypoxic brain injury.^6^, ^7^, ^8^, ^9^, ^10^, ^11^ Fetal blood sampling (FBS) has been shown to reduce operative vaginal delivery rates without affecting neonatal outcomes, but is a complex, invasive procedure.^6^, ^12^ More accurate intrapartum surveillance methods are needed to decrease adverse neonatal outcomes while minimizing obstetric intervention. Fetal pulse oximetry (FPO) may improve intrapartum fetal evaluation by providing a non‐invasive measurement of fetal oxygen saturation (FSpo_2_).^13^, ^14^, ^15^, ^16^

A narrative review by Uchida et al.^17^ concluded that measuring FSpo_2_ with FHR monitoring in cases of non‐reassuring fetal status may reduce cesarean section (CS) rates.

A Cochrane Review compared intrapartum FSpo_2_ monitoring with other fetal surveillance techniques across seven trials (8013 pregnancies).^18^ No significant differences were noted in the overall CS rate between those that monitored FSpo_2_ and those that did not monitor FSpo_2_ or for which the FSpo_2_ results were not displayed to the clinician or woman (four studies, *n* = 4008, risk ratio [RR] 0.99, 95% confidence intervals [CI] 0.86–1.13). This review examined the association between FSpo_2_ monitoring and birth and neonatal outcomes, but did not directly consider the association of low FSpo_2_ with adverse perinatal and long‐term neonatal outcomes. Therefore, the objective of this systematic review and meta‐analysis was to take a more comprehensive, up‐to‐date approach, incorporating evidence not just from randomized controlled trials (RCTs), but also from non‐randomized studies, examining the association between intrapartum fetal oxygen saturation and perinatal and long‐term neurodevelopmental outcomes. The null hypothesis is that an FSpo_2_ less than 30% is not associated with adverse perinatal and long‐term neurodevelopmental outcomes and that the addition of FSpo_2_ monitoring to traditional monitoring does not impact birth, perinatal, or long‐term neurodevelopmental outcomes.

## 2 MATERIALS AND METHODS

### 2.1 Review questions

This review aimed to answer the following two questions:

1. Is intrapartum fetal oxygen saturation less than 30% associated with an increased risk of adverse perinatal and long‐term neurodevelopmental outcomes?
2. Does the addition of fetal pulse oximetry to established forms of fetal monitoring reduce the operative delivery rate without affecting perinatal and long‐term neurodevelopmental outcomes?

### 2.2 Eligibility criteria

The following PICO (Patient, Intervention, Comparison, and Outcome) criteria guided this systematic review.

The Population was women in labor with a cephalic position baby. Intervention/Exposure were (1) low fetal oxygen saturation defined as less than 30% (exposure) and (2) the use of fetal pulse oximetry during labor to measure intrapartum fetal oxygen saturation (intervention).

Comparison was between (1) normal fetal oxygen saturation defined as greater than 30% (unexposed group) and (2) traditional fetal monitoring, e.g. FHR monitoring by CTG or fetal scalp electrode, fetal blood sampling or fetal electrocardiogram (ECG), which was compared to traditional fetal monitoring without measuring fetal oxygen saturation (control). Notably, we elected to include all studies using a 30% FSpo_2_ threshold, whether defined as less than 30% or 30% or less, to avoid excluding valuable data. In the results, we documented the FSpo_2_ threshold used in each study. We conducted subgroup analyses, excluding the studies with the inclusive 30% or less criterion, to assess if this small change in definition altered the results. We have documented “abnormal FSpo_2_” as less than 30% and normal FSpo_2_ as more than 30% for readability.

The primary Outcomes of this review were: (1) umbilical artery (UA) pH less than 7.20, (2) UA pH less than 7.15, (3) UA pH less than 7.0, and (4) 5‐min APGAR Score less than 7. Secondary outcomes included: (1) UA base excess −10 mmol/L or less, (2) UA lactate more than 4.8 mmol/L, (3) admission to the neonatal intensive care unit (NICU), (4) neonatal or intrapartum death, (5) cardiopulmonary resuscitation or intubation required within 24 h of delivery, (6) hypoxic ischemic encephalopathy, (7) umbilical vein oxygen saturation less than 55%, (8) UA oxygen saturation less than 30%, (9) operative delivery for non‐reassuring fetal status (as defined by local protocols in each study), (10) operative delivery for dystocia, (11) fetal scalp pH less than 7.20, (12) fetal scalp lactate more than 4.8 mmol/L, (13) cerebral palsy, and (14) severe neurodevelopmental disability.^19^

We included RCTs, non‐randomized control trials, and observational studies including cohort and case–control studies.

Our review exclusion criteria included: (1) studies only available in abstract form, (2) non‐human studies, (3) review articles, case reports, and case series, and (4) conference proceedings, letters, commentaries, notes, editorials, and dissertations.

### 2.3 Literature search

This review was conducted according to the Preferred Reporting Items for Systematic Reviews and Meta‐Analyses (PRISMA) and Meta‐Analysis of Observational Studies in Epidemiology (MOOSE) guidelines (Appendices S2 and S3).^20^, ^21^ The protocol was prospectively registered in PROSPERO (registration number CRD42023457368) and published.^22^

The search strategy was developed with librarian assistance. We conducted a comprehensive search of PubMed, EMBASE, CINAHL, The Cochrane Library, Web of Science, ClinicalTrials.gov, and WHO ICTRP from database inception through February 2024, with no restrictions on date, geographic region, country income level, or language (Appendix S1). Corresponding authors of ongoing trials were contacted by email to request available results. Our search was supplemented by hand‐searching reference lists of included articles.

### 2.4 Study screening and selection

Three clinician reviewers (JM, SW, LOB) individually screened all titles and abstracts. Full texts were obtained where necessary to screen for eligibility. Discrepancies were resolved through a fourth review author (FMcC). Translations of non‐English were obtained using DeepL and colleagues proficient in the relevant languages.^23^

### 2.5 Data extraction

Two clinician authors (JM, SW) independently extracted data using a standardized, pre‐piloted data extraction form. Discrepancies were resolved through discussion with a third author (FMcC).

### 2.6 Quality appraisal and bias

Articles were assessed for methodologic quality independently by two reviewers (JM and SW) using the Cochrane Risk of Bias tool^24^ for RCTs and the Newcastle‐Ottawa Scale^25^ for observational studies. Disagreements were resolved by discussion with a third reviewer (FMcC).

Publication bias was evaluated using funnel plots and Egger test, for meta‐analyses with at least 10 studies.

The grading of recommendations, assessment, development, and evaluation (GRADE) approach was used to evaluate evidence certainty, with two reviewers (JM, SW) independently using GRADEpro software.^26^

### 2.7 Data synthesis, including assessment of heterogeneity

A random effect meta‐analysis was conducted for each exposure‐outcome association using Revman Web.^27^ We intended to use the generic inverse variance method to analyze both crude and adjusted estimates.

Relevant and comparable studies were included in a combined meta‐analysis but we did not combine randomized and non‐randomized studies. Subgroup analyses differentiated between non‐randomized study types. Summary measures were reported as odds ratios (OR) with 95% CI. ORs were calculated using raw data based on outcome counts within intervention and comparison groups. Correlations between FSpo_2_ levels and umbilical artery pH, and FSpo_2_ levels and umbilical vein pH were presented narratively. Heterogeneity was assessed using the *I* ^2^ statistic and explored through subgroup analyses by study design and quality. For meta‐analyses with at least three studies, 95% prediction intervals were calculated.^28^, ^29^ Subgroup analyses excluded studies that did not standardize delivery decisions based on FSpo_2_ values and those comparing FSpo_2_ with FHR and fetal ECG monitoring. Additional subgroup analyses were also conducted based on gestational age and timing of low FSpo_2_. As discussed previously, we conducted subgroup analyses, excluding studies with the inclusive 30% or less criterion to assess if this small definitional change affected results.

## 3 RESULTS

### 3.1 Search results

The search strategy identified 3280 articles. After removing duplicates, 2042 studies remained (Appendix S1, Table S1). Following title and abstract screening, 205 full‐text articles were reviewed, and 162 studies were excluded for not meeting the inclusion criteria. This resulted in 47 included papers, comprising 13 071 mother‐infant pairs: 7925 from RCTs, 218 from non‐RCTs, 4928 from cohort studies, and 67 from case–control studies. The characteristics and populations of the included studies are detailed in Tables S1 and S2 of Appendix S5.

### 3.2 Association between fetal oxygen saturation less than 30% and adverse neonatal outcomes

#### Umbilical artery pH levels

**FIGURE 1:**
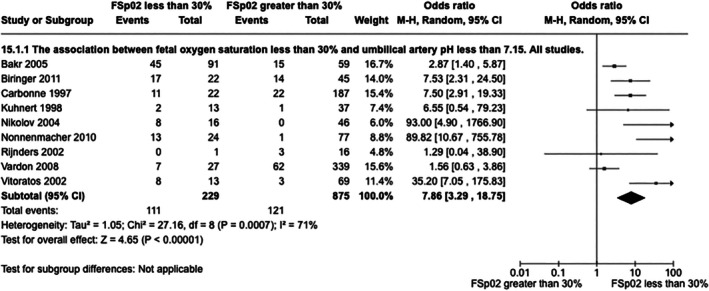
Association between fetal oxygen saturation less than 30% and umbilical artery pH less than 7.15. All studies.

FSpo_2_ less than 30% was associated with UA pH less than 7.15 (eight cohort studies, one case–control study, 229 low FSpo_2_ cases, 875 cases with FSpo_2_ >30%, OR 7.86, 95% CI 3.29–18.75, *I* ^2^ = 71%, *P* = 0.001, Figure 1).^30^, ^31^, ^32^, ^33^, ^34^, ^35^, ^36^, ^37^, ^38^ Similarly, an association between FSpo_2_ less than 30% and UA pH less than 7.20 was noted (11 cohort studies, 209 FSpo_2_ <30% and 723 >30%, OR 5.53, 95% CI 2.17–14.07, *I* ^2^ = 66%, *P* < 0.001, Appendix S7, Figure S1).^33^, ^36^, ^39^, ^40^, ^41^, ^42^, ^43^, ^44^, ^45^, ^46^, ^47^ McNamara et al.^48^ was excluded from this analysis as it reported no instances of FSpo_2_ less than 30%.

#### 5‐min Apgar score <7

**FIGURE 2:**
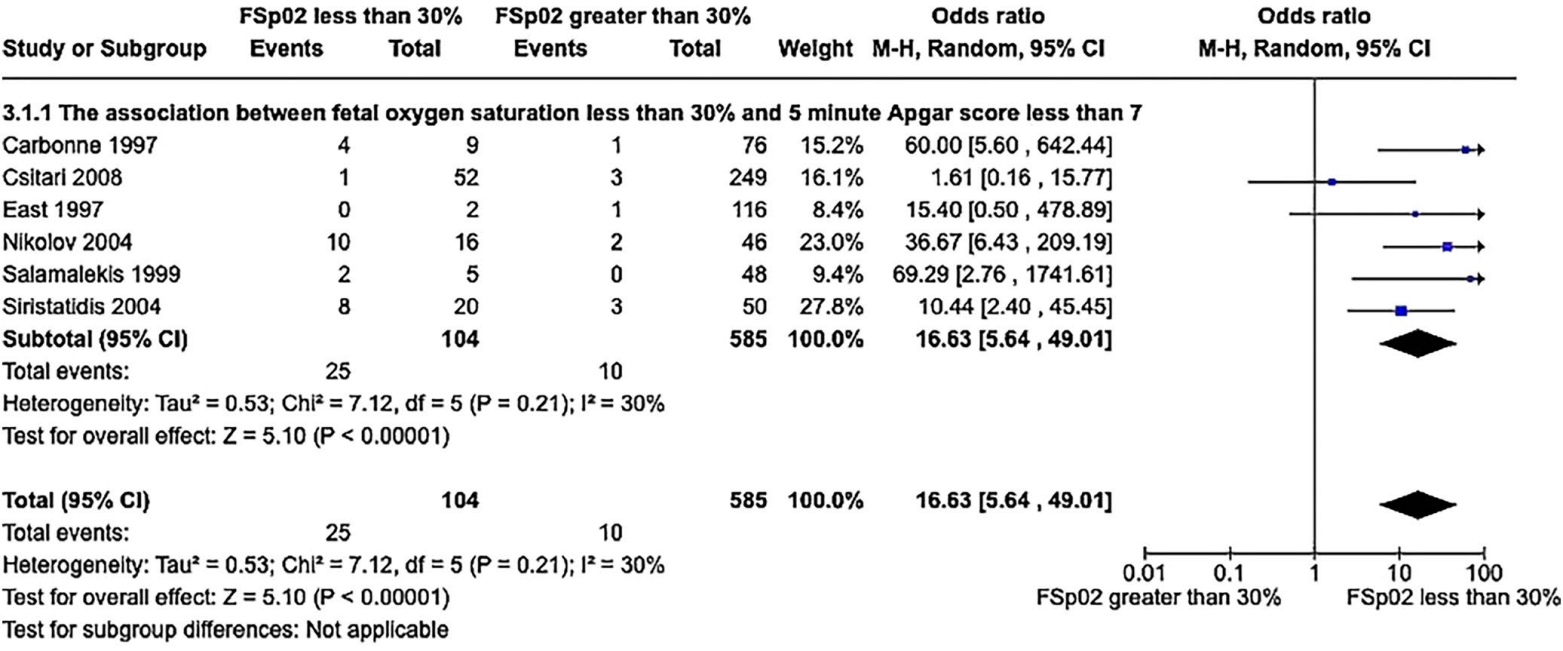
Association between fetal oxygen saturation less than 30% and 5‐min Apgar score less than 7.

FSpo_2_ less than 30% was associated with 5‐min Apgar scores less than 7 (6 cohort studies, 104 cases of FSpo_2_ <30% and 585 cases of FSpo_2_ >30%, OR 16.63, 95% CI 5.64–49.01, *P* < 0.001, *I* ^2^ = 30%, Figure 2).^32^, ^34^, ^40^, ^41^, ^43^, ^45^, ^46^, ^49^, ^50^ The study by Langer et al.^32^ was excluded from the analysis as no infants had a 5‐min Apgar score less than 7.

#### Neonatal intensive care unit admissions

A significant association was noted between FSPo_2_ less than 30% and admissions to the NICU or special care baby unit (3 cohort studies, 49 neonates with FSpo_2_ <30% and 207 with FSpo_2_ >30%, OR 5.89, 95% CI 1.73–20.01, *P* = 0.005, *I* ^2^ = 0%, Appendix S7, Figure S2).^34^, ^46^, ^50^ The analysis excluded the study by Csitari et al.^40^ because of the absence of any infant admissions to the NICU in either the exposed or non‐exposed groups.

### 3.3 Association between the addition of fetal oxygen saturation monitoring to fetal heart rate monitoring and birth outcomes

#### Operative delivery rates

The use of FSpo_2_ monitoring in addition to FHR monitoring ± FBS or fetal ECG was not associated with a reduction in CS compared with traditional monitoring without FSpo_2_ (8 RCTs, 3914 cases of FSpo_2_ monitoring, 4011 cases without FSpo_2_ monitoring, OR 0.79, 95% CI 0.58–1.07, *P* = 0.130, *I* ^2^ = 79%, Appendix S7, Figure S3).^42^, ^51^, ^52^, ^53^, ^54^, ^55^, ^56^, ^57^, ^58^, ^59^ However, a subgroup analysis, excluding Bloom et al.^51^ due to its allowance for individual clinical management decisions rather than standardizing delivery expedience based on FSpo_2_ values, and excluding Valverde et al.^59^ for comparing FPO with FHR and fetal ECG monitoring, rather than FPO with FHR monitoring against FHR monitoring alone with or without the use of FBS, found a lower CS rate with FSpo_2_ monitoring (six RCTs with 1195 cases with FSpo_2_ monitoring and 1209 without FSpo_2_ monitoring, OR 0.61, 95% CI 0.39–0.96, *I* ^2^ = 81%, *P* = 0.030, see Appendix S9, Figure S8).

#### Operative delivery rates for non‐reassuring fetal status

**FIGURE 3:**
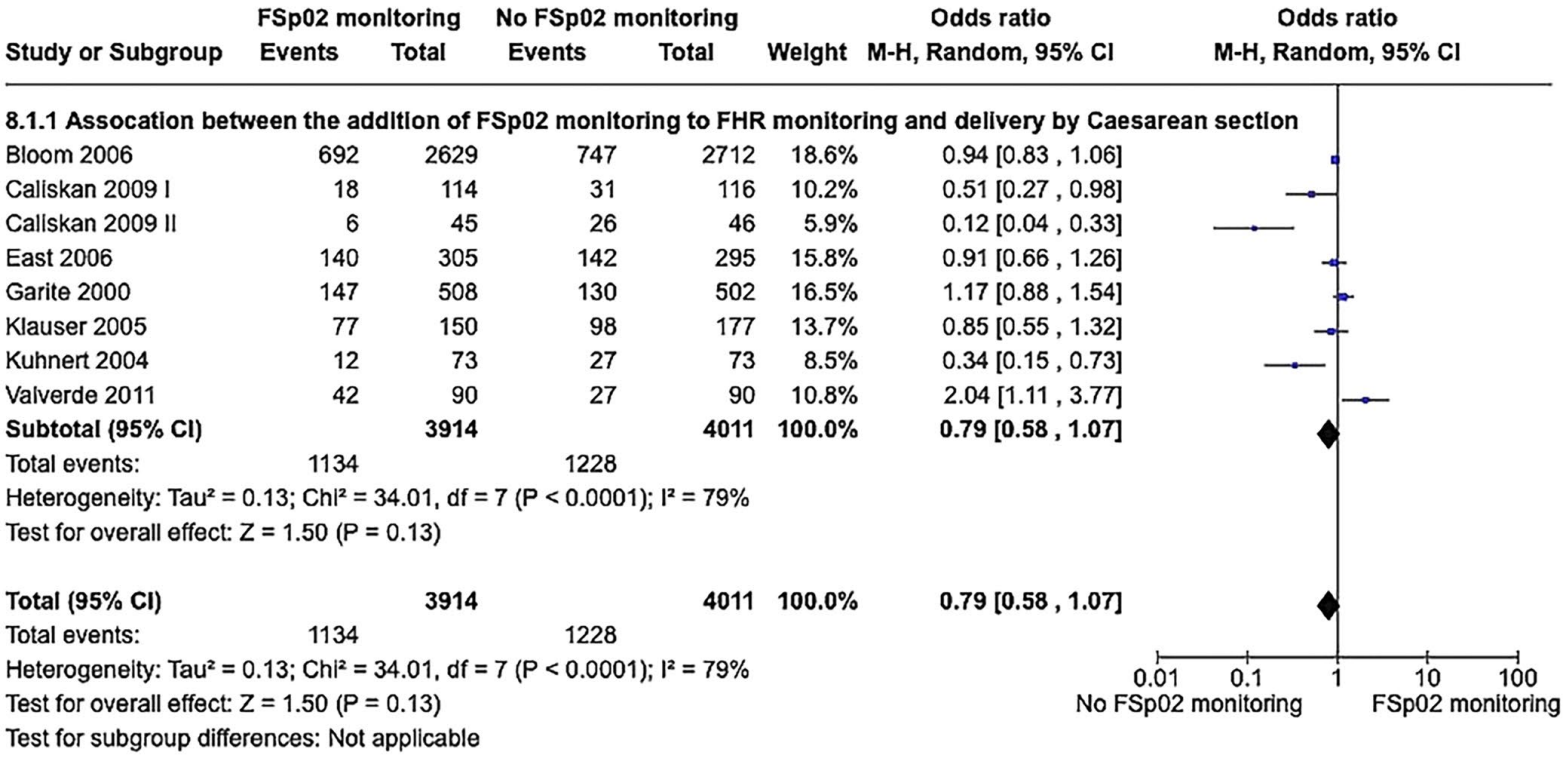
Association between addition of fetal oxygen saturation monitoring to fetal heart rate monitoring and cesarean sections for non‐reassuring fetal status.

The addition of FSpo_2_ monitoring to FHR monitoring was associated with a lower odds of CS performed for non‐reassuring fetal status (8 RCTs, OR 0.59, 95% CI 0.40–0.86, *P* = 0.006, *I* ^2^ = 71%, Figure 3).^51^, ^52^, ^53^, ^54^, ^56^, ^57^, ^58^, ^59^ Operative vaginal delivery rates for non‐reassuring fetal status were not influenced by the addition of FSpo_2_ monitoring to standard monitoring (Appendix S7, Figure S4; 4 RCTs, 915 cases of FSpo_2_ monitoring + FHR, 861 cases of FHR without FSpo_2_ monitoring, OR 0.45, 95% CI 0.17–1.18, *P* = 0.100, *I* ^2^ = 87%).^54^, ^56^, ^58^, ^59^

#### Operative delivery rates for dystocia in labor

The use of FSpo_2_ monitoring in conjunction with FHR monitoring did not alter the incidence of CS for dystocia compared with FHR monitoring alone (8 RCTs, 3866 cases of FSpo_2_ + FHR, 3948 cases of FHR without FSpo_2_, OR 1.07, 95% CI 0.95–1.20, *P* = 0.270, *I* ^2^ = 73%, Appendix S7, Figure S5).^51^, ^52^, ^53^, ^54^, ^56^, ^57^, ^58^, ^59^ Conversely, the addition of FSpo_2_ monitoring was associated with an increased odds of operative vaginal deliveries for dystocia in the second stage of labor (3 RCTs, 407 cases of FSpo_2_ + FHR, 400 cases of FHR without FSpo_2_, OR 1.97, 95% CI 1.18–3.29, *I* ^2^ = 0%, *P* = 0.009, Appendix S7, Figure S6).^54^, ^58^, ^59^

### 3.4 The association between the addition of fetal oxygen saturation monitoring to fetal heart rate monitoring and neonatal outcomes

#### Umbilical artery pH levels

**FIGURE 4:**
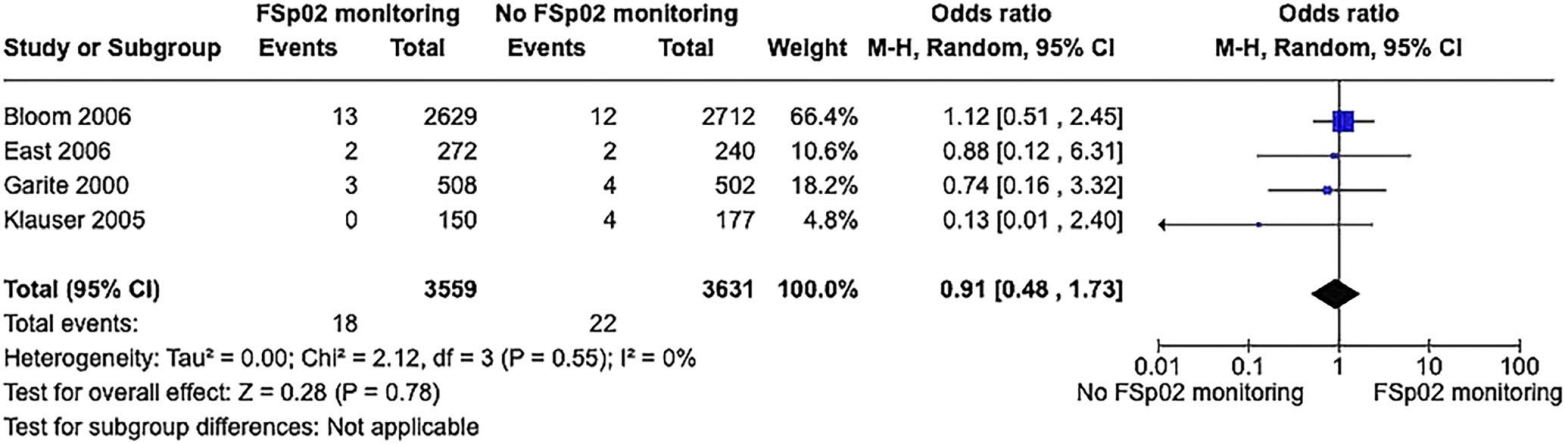
Association between addition of fetal oxygen saturation monitoring to fetal heart rate monitoring and umbilical artery pH less than 7.0.

No significant differences in UA pH levels were found when FSpo_2_ monitoring was added to FHR monitoring ± FBS. The UA pH levels remained stable in neonates with normal FSpo_2_ levels even when delivery was not expedited in response to non‐reassuring fetal heart rate patterns. For UA pH less than 7.15 (3 RCTs, 431 with FSpo_2_ + FHR versus 402 without FSpo_2_, OR 0.90, 95% CI 0.50–1.64, *P* = 0.730, *I* ^2^ = 10%, Appendix S7, Figure S7), and for UA pH less than 7.0 (4 RCTs, 3559 with FSpo_2_ + FHR versus 3631 without FSpo_2_, OR 0.91, 95% CI 0.48–1.73, *P* = 0.780, *I* ^2^ = 0%, Figure 4).^51^, ^56^, ^57^, ^60^

#### Umbilical artery base excess levels

No significant differences in UA base excess levels of −10 mmol/L or less were found when FSpo_2_ monitoring was used as well as FHR monitoring ± FBS. The UA base excess levels remained stable in neonates with normal FSpo_2_ levels even when delivery was not expedited in response to non‐reassuring fetal heart rate patterns (3 RCTs, FSpo_2_ + FHR monitoring: 499 cases, no FSpo_2_: 448 cases, OR 1.26, 95% CI 0.81–1.95, *I* ^2^ = 0%, *P* = 0.310, Appendix S7, Figure S8).^52^, ^54^, ^56^

#### 5‐min Apgar score less than 7

The addition of FSpo_2_ to FHR monitoring was not associated with a difference in 5‐min Apgar scores less than 7 (5 RCTs, 692 infants with FSpo_2_ + FHR ± FBS and 742 FHR ± FBS, OR 0.66, 95% CI 0.37–1.17, *I* ^2^ = 0%, *P* = 0.160, Appendix S7, Figure S9).^52^, ^53^, ^56^, ^57^, ^60^ The 5‐min Apgar score remained stable in cases where infants, displaying normal FSpo_2_ levels, were not expedited for delivery in the context of non‐reassuring FHR patterns.

#### Admission to the neonatal intensive care unit

The rates of admission were not increased when delivery was not expedited for non‐reassuring FHR patterns in the setting of normal FSpo_2_ (5 RCTs, 3706 neonates with FSpo_2_ monitoring, 3802 without FSpo_2_ monitoring, OR 0.98, 95% CI 0.82–1.18, *I* ^2^ = 0%, *P* = 0.840), Appendix S7, Figure S10.^51^, ^52^, ^56^, ^57^, ^60^

#### Neonatal critical care interventions

Across five RCTs involving 5757 neonates, 2821 infants were monitored with both FSpo_2_ and FHR (with or without fetal blood sampling), while 2936 received traditional FHR monitoring (with or without fetal blood sampling). When delivery was not expedited despite non‐reassuring FHR patterns but normal FSpo_2_, there was no significant difference in neonatal intubation rates between the two groups (OR 0.89, 95% CI 0.54–1.46, *I* ^2^ = 0%, *P* = 0.590, Appendix S7, Figure S11).^51^, ^52^, ^56^ The associations between the addition of FSpo_2_ monitoring to FHR monitoring and cardiopulmonary resuscitation, hypoxic ischemic encephalopathy, and neonatal or intrapartum death are discussed in Appendix S7.

### 3.5 The association between the addition of fetal oxygen saturation monitoring to fetal heart rate monitoring and long‐term outcomes

One study investigated the association between the addition of FSpo_2_ to FHR monitoring in labor and long‐term outcomes. It did not meet our criteria for severe neurodevelopmental disability.^19^ Neurodevelopmental, motor, and behavioral outcomes were compared across three groups: children with abnormal CTG and normal FSpo_2_ (Group 1, *n* = 32), abnormal CTG delivered by CS without FSpo_2_ monitoring (Group 2, *n* = 25), and spontaneous births without signs of hypoxia (Group 3, *n* = 31). Motor development scores were lower in Group 1 (91.8 ± 11.5) than in Group 3 (98.6 ± 12.1, *t* = 2.395, *P* < 0.050), with no difference between Group 1 and 2. Group 3 had better overall behavior and interest than Group 1 (overall behavior: Group 1: 65.9 ± 22.4, Group 3: 78.5 ± 15.5, *t* = 2.586, *P* < 0.050, interest: Group 1: 65.2 ± 22.9, Group 3: 76.2 ± 15.7, *t* = 2.205, *P* < 0.050).

### 3.6 Additional analyses

Appendix S7 discusses the correlation between FSpo_2_ and UA pH, umbilical vein pH, and the association between FSpo_2_ less than 30% and UA lactate levels. It also addresses the association between FSpo_2_ and umbilical cord oxygen saturation levels, fetal scalp pH levels, and the impact of adding FSpo_2_ monitoring to FHR monitoring on UA lactate levels and operative delivery rates. Additionally, it outlines the methods used to measure FSpo_2_ in the included studies.

### 3.7 Quality assessment of included studies

The quality of the reviewed studies was inconsistent, as detailed in Appendix S8, Tables S1 and S2. Among the observational studies, 13 (37.2%) were rated “fair quality” and 25 (65.8%) as “good quality”. For the RCTs, one study (12.5%) was “fair quality” and seven (87.5%) were “poor quality” (see Appendix S8, Figures S1 and S2 and Tables S1 and S2 for details on the percentage risk level per domain across all studies and the review authors' judgments about each risk of bias item, respectively). As a result of the poor quality of most RCTs, no subgroup analyses based on quality were performed for them. Subgroup analyses for observational studies are described in Appendix S9.

### 3.8 Grading of recommendations, assessment, development, and evaluation certainty of the evidence

The evidence for the association between low FSpo_2_ and adverse perinatal outcomes was rated as “low” to “moderate” (non‐randomized studies). This rating was downgraded due to study design, significant risk of bias (Newcastle‐Ottawa Scale), inconsistency (variance of point estimates), and imprecision (wide 95% CI). For the addition of FSpo_2_ monitoring to standard monitoring, the evidence was consistently rated as “very low” (RCTs). This was because of the significant risk of bias (low Risk of Bias scores), inconsistency (wide point estimate variances), and imprecision (broad 95% CI crossing clinical decision thresholds). Details are given in Appendix S8, Tables S3 and S4.

### 3.9 Subgroup analysis and assessment of heterogeneity

Of the 47 included studies, 23 contained data comparing perinatal outcomes in those with normal FSpo_2_ levels and “abnormal” FSpo_2_ levels. Twenty studies (86.96%) defined abnormal FSpo_2_ as less than 30%, whereas, 3 studies (13.04%) defined abnormal FSpo_2_ as 30% or less. Full study details are provided in Appendix S5. Subgroup analyses were performed where studies that defined abnormal FSpo_2_ as 30% or less were excluded from the analyses.^30^, ^34^, ^45^ These analyses are documented in Appendix S9, Figures S19 to S21, but the results did not differ significantly from the primary analyses.

Subgroup analyses based on gestational age, methodologic heterogeneity, and study quality did not materially alter interpretation of results (Appendix S9). The 95% prediction intervals for all analyses are presented in Appendix S10, Tables S1 and S2. Funnel plots and Egger test showed no significant evidence of publication bias (Appendix S7).

### 3.10 Timing of low fetal oxygen saturation

There is no agreed threshold for duration of low FSpo_2_. The included studies specified that duration of low FSpo_2_ varied as documented in detail in Appendix S5, Table S1. The most common duration was FSpo_2_ less than 30% for a continuous period of 10 min or more, with 15 studies adopting that criterion and one further study adopting a definition of FSpo_2_ less than 30% for a cumulative period of 10 min or more. The second most commonly used criterion was average FSpo_2_ less than 30% for 30 min (seven studies). Subgroup analysis based on timing of low FSpo_2_ is described in Appendix S9.

## 4 DISCUSSION

### 4.1 Main findings

This systematic review and meta‐analysis synthesized the published literature examining the association between intrapartum FSpo_2_ and adverse perinatal and long‐term neurodevelopmental outcomes. FSpo_2_ less than 30% was associated with umbilical artery pH less than 7.20 and 7.15, 5‐min Apgar scores less than 7, and NICU admission. The addition of FSpo_2_ monitoring to traditional intrapartum monitoring was associated with fewer CS for suspected fetal compromise in labor without increasing perinatal morbidity. There was no association between umbilical artery pH less than 7.15 or 7.0 and the addition of FSpo_2_ monitoring to FHR monitoring. Similarly, the integration of FSpo_2_ monitoring was associated with no difference in 5‐min Apgar scores less than 7, umbilical artery base excess of −10 mmol/L or less, admission to the NICU, or neonatal intubation. FSpo_2_ correlated strongly with fetal scalp pH. Measuring FSpo_2_ is also far less invasive and is technically simpler than obtaining fetal blood samples. One study investigated the association between the addition of FSpo_2_ to FHR monitoring in labor and long‐term outcomes, but it did not meet our criteria for severe neurodevelopmental disability or disclose the FSpo_2_ threshold used.^19^ Future research is needed to evaluate long‐term outcomes. Also, we could not perform a meta‐analysis to assess the addition of FSpo_2_ monitoring to fetal heart rate monitoring and cardiopulmonary resuscitation, hypoxic ischemic encephalopathy and neonatal or intrapartum death because of limited data. Subgroup analyses based on gestational age, methodologic heterogeneity, and study quality did not materially alter the interpretation of results.

### 4.2 Interpretation

Our results suggest that FSpo_2_ monitoring can be an effective adjunct to standard FHR monitoring, significantly reducing the odds of CS for non‐reassuring fetal status without increasing the risk of negative neonatal consequences.

FSpo_2_ was widely used in the 1990s but its use declined after a 2006 RCT by Bloom et al.^51^ found no reduction in CS rates with FSpo_2_ monitoring. This study had limitations, including variability in clinical practices and a brief threshold for low FSpo_2_.^18^ This study impacted the systematic review by East et al.,^18^ which concluded that combining FPO with FHR monitoring did not decrease the overall rate of CS. We noted a lower odds of CS in the FSpo_2_ group, after excluding studies that did not standardize delivery decisions based on FSpo_2_ values and those comparing FPO with FHR and fetal ECG monitoring.

The present review noted the predominant use of the Nellcor device in the included studies. The Nellcor device was inserted transcervically with its sensor against the fetal cheek or temple, the device emitted red and infrared light, detected the reflected signal, and displayed the calculated FSpo_2_ and pulse rate on a monitor.^61^ Positioned between the fetal cheek or temple and the uterine wall, it may theoretically impede fetal head rotation and increase dystocia risk.^62^ This device has also been critiqued for suboptimal signal quality, with many studies reporting signal qualities of less than 75% (Appendix S7).^35^, ^38^, ^62^

### 4.3 Future research

Future research should prioritize developing an optimized FPO device, defining the duration for which FSpo_2_ less than 30% necessitates delivery, and should include long‐term neurodevelopmental outcomes. Although our findings support FPO as a helpful adjunct in intrapartum monitoring, evaluating its ability to replace established diagnostic measures such as FBS lies beyond the scope of this review and should be addressed in dedicated comparative diagnostic studies.

### 4.4 Strengths and limitations

This systematic review has several strengths. First, it provides a comprehensive synthesis of research on the association between intrapartum FPO and adverse neonatal outcomes. We included multiple study types because observational studies are more likely to explore the association between low FSpo_2_ and adverse neonatal outcomes. In contrast, RCTs are more likely to assess the impact of adding FSpo_2_ monitoring to traditional methods on neonatal and birth outcomes. This approach provides a thorough evaluation of FSpo_2_ monitoring. Second, the review's robustness is enhanced by implementing a comprehensive search strategy, a prospectively registered protocol, and adherence to PRISMA and MOOSE guidelines.^21^, ^63^ Including clinical trial registers aimed to reduce publication bias. Funnel plots and Egger test showed no significant evidence of such bias. Third, involving three reviewers for eligibility screening and two for data extraction and quality assessment reduces potential reviewer bias. Lastly, the absence of language restrictions minimizes the likelihood of missing relevant studies.

There are also limitations, including limitations of the current literature. First, although we planned to use the generic inverse variance method to analyze crude and adjusted estimates for each exposure‐outcome association, previous studies did not adjust for potential confounders. However, this may be less of an issue with estimates from RCTs. Consequently, our meta‐analysis was confined to the synthesis of crude estimates only. Second, many of our computed meta‐analysis prediction intervals were notably wide. Results should be interpreted with caution given the presence of heterogeneity between studies. Third, the definitions of the terms “abnormal” CTG and “non‐reassuring fetal status” varied across studies, potentially causing inconsistencies. However, these were based on hospital or trial policies, suggesting that they were appropriately defined within their contexts. Fourth, family‐wise error rates increase with multiple comparisons of secondary outcomes, so significant results should be interpreted cautiously, considering plausibility, theory, and uncertainty. Fifth, only one study examined long‐term outcomes in children monitored with intrapartum FPO.^64^ This study did not specify the FSpo_2_ level for deciding whether to expedite delivery or continue labor, nor the criteria for these decisions. Sixth, while our systematic review employed a comprehensive search strategy without restrictions on country or income level, most included studies (*n* = 39, 83%) originated from high‐income settings. Consequently, the applicability of our findings to low‐resource regions may be reduced. Lastly, the evidence for the association between low FSpo_2_ and adverse perinatal outcomes was rated as “low” to “moderate” for non‐randomized studies. For the addition of FSpo_2_ monitoring to standard methods, the evidence from RCTs was rated as “very low”. These ratings must be considered when interpreting results.

In conclusion, FSpo_2_ less than 30% is associated with adverse perinatal outcomes, supporting its potential as a valuable adjunct in intrapartum monitoring. FSpo_2_ combined with traditional fetal heart rate monitoring may reduce unnecessary intrapartum cesareans for suspected fetal distress without affecting short‐term neonatal outcomes. Further study is needed to understand long‐term implications.

## AUTHOR CONTRIBUTIONS

JMM contributed to conceptualization, investigation, methodology, visualization, and writing—original draft preparation; SW and LO'B contributed to validation and writing—review & editing; VC contributed to the investigation and methodology; RB contributed to conceptualization, methodology, and writing—review & editing; and ASK, JH, RG, GMM, and FPM contributed to conceptualization, methodology, supervision, and writing—review & editing.

## CONFLICT OF INTEREST STATEMENT

The authors have no conflicts of interest.

## Supporting information


**Appendix S1.** Search strategy.
**Appendix S2.** Moose checklist for meta‐analyses of observational studies.
**Appendix S3.** PRISMA 2020 checklist.
**Appendix S4.** PRISMA diagram.
**Appendix S5.** Details of included studies.
**Appendix S6.** Correlation between fetal oxygen saturation and umbilical cord pH values.
**Appendix S7.** Additional data.
**Appendix S8.** Quality assessment and GRADE analysis.
**Appendix S9.** Analyses.
**Appendix S10.** Prediction intervals.

## Data Availability

All data supporting this study are available upon request.

## References

[1] Chandraharan E , Wiberg N . Fetal scalp blood sampling during labor: an appraisal of the physiological basis and scientific evidence. Acta Obstet Gynecol Scand. 2014;93:544‐547. doi:10.1111/aogs.12416 24806702

[2] The Royal Australian and New Zealand College of Obstetricians and Gynaecologists (RANZCOG) . Intrapartum Fetal Surveillance, Clinical Guideline, Fourth Edition. 2019.

[3] HNWaIHPob and the Fetal Heart Rate Monitoring Working Group . National Clinical Guideline for Fetal Heart Rate Monitoring. Health Service Executive; 2019.

[4] Ayres‐de‐Campos D , Spong CY , Chandraharan E , et al. FIGO consensus guidelines on intrapartum fetal monitoring: Cardiotocography. Int J Gynaecol Obstet. 2015;131:13‐24. doi:10.1016/j.ijgo.2015.06.020 26433401

[5] National Institute for Health and Care Excellence (NICE) . Intrapartum Care (NG235). 2023.

[6] Alfirevic Z , Devane D , Gyte G . Continuous cardiotocography (CTG) as a form of electronic fetal monitoring (EFM) for fetal assessment during labour. Cochrane Database Syst Rev. 2006;3. 10.1002/14651858.CD006066 16856111

[7] Vintzileos AM , Antsaklis A , Varvarigos I , et al. A randomized trial of intrapartum electronic fetal heart rate monitoring versus intermittent auscultation. Obstet Gynecol. 1993;81:899‐907. 8497353

[8] MacDonald D , Grant A , Sheridan‐Pereira M , Boylan P , Chalmers I . The Dublin randomized controlled trial of intrapartum fetal heart rate monitoring. Am J Obstet Gynecol. 1985;152(5):524‐539. doi:10.1016/0002-9378(85)90619-2 3893132

[9] Kelso IM , Parsons RJ , Lawrence GF , Arora SS , Edmonds DK , Cooke ID . An assessment of continuous fetal heart rate monitoring in labor. A randomized trial. Am J Obstet Gynecol. 1978;131:526‐532. doi:10.1016/0002-9378(78)90114-x 677195

[10] Grant A , O'Brien N , Joy MT , Hennessy E , MacDonald D . Cerebral palsy among children born during the Dublin randomised trial of intrapartum monitoring. Lancet. 1989;2:1233‐1236. doi:10.1016/s0140-6736(89)91848-5 2573757

[11] Grivell RM , Alfirevic Z , Gyte GM , Devane D . Antenatal cardiotocography for fetal assessment. Cochrane Database Syst Rev. 2015;2015(9):Cd007863. doi:10.1002/14651858.CD007863.pub4 26363287 PMC6510058

[12] Jørgensen J , Weber T . Fetal scalp blood sampling in labor—a review. Acta Obstet Gynecol Scand. 2014;93:548‐555. 24806978 10.1111/aogs.12421

[13] Yam J , Selina C , Arulkumaran S . Intrapartum fetal pulse oximetry. Part I: principles and technical issues. Obstet Gynecol Surv. 2000;55:163‐172. 10713982 10.1097/00006254-200003000-00025

[14] Colditz P , Begg L , East C . Fetal pulse oximetry: instrumentation and recent clinical experience. Clin Perinatol. 1999;26:869‐880. 10572726

[15] Kuhnert M , Seelbach‐Gobel B , Di Renzo G , et al. Guidelines for the use of fetal pulse oximetry during labor and delivery. Prenat Neonatal Med. 1999;3:432‐433.

[16] East CE , Colditz PB , Begg LM , Brennecke SP . Update on intrapartum fetal pulse oximetry. Aust N Z J Obstet Gynaecol. 2002;42(2):119‐124. doi:10.1111/j.0004-8666.2002.00119.x 12069136

[17] Uchida T , Kanayama N , Kawai K , et al. Reevaluation of intrapartum fetal monitoring using fetal oximetry: a review. J Obstet Gynaecol Res. 2018;44:2127‐2134. doi:10.1111/jog.13761 30084196

[18] East CE , Begg L , Colditz PB . Fetal pulse oximetry for fetal assessment in labour. Cochrane Database Syst Rev. 2014;10:CD004075. 10.1002/14651858.CD004075.pub4 PMC7104297 25287809

[19] Devane D , Lalor JG , Daly S , McGuire W , Cuthbert A , Smith V . Cardiotocography versus intermittent auscultation of fetal heart on admission to labour ward for assessment of fetal wellbeing. Cochrane Database Syst Rev. 2017;1(1):CD005122. doi:10.1002/14651858.CD005122.pub5 28125772 PMC6464914

[20] Howells E . Peri‐operative management of a pregnancyrelated hiatus hernia complication. Anaesthesia. 2018;73:18. Conference Abstract. doi:10.1111/anae.14448

[21] Stroup DF , Berlin JA , Morton SC , et al. Meta‐analysis of observational studies in epidemiology: a proposal for reporting. JAMA. 2000;283(15):2008‐2012. doi:10.1001/jama.283.15.2008 10789670

[22] Mitchell J , Walsh S , O'Byrne L , et al. Association between intrapartum fetal pulse oximetry and adverse perinatal and long‐term outcomes: a systematic review and meta‐analysis protocol. HRB Open Res. 2024;6:63. doi:10.12688/hrbopenres.13802.2 38628596 PMC11019289

[23] Kutylowski J . DeepL. 2018 Accessed December 10, 2023. https://www.deepl.com/translator

[24] Higgins J , Altman D , Gøtzsche P , et al. The Cochrane Collaboration's tool for assessing risk of bias in randomised trials. BMJ. 2011;343:d5928. 22008217 10.1136/bmj.d5928 PMC3196245

[25] Wells G , Shea B , O'Connell D , et al. The Newcastle–Ottawa Scale (NOS) for assessing the quality of non‐randomized studies in meta‐analysis. Ottawa Hospital Research Institute; 2000.

[26] Balshem H , Helfand M , Schünemann HJ , et al. GRADE guidelines: 3. Rating the quality of evidence. J Clin Epidemiol. 2011;64:401‐406. doi:10.1016/j.jclinepi.2010.07.015 21208779

[27] Review Manager Web (RevMan Web) [Computer program] . The Cochrane Collaboration. 2022, Accessed April 1 2024. https://revman.cochrane.org

[28] Borenstein M , Higgins JP , Hedges LV , Higgins JPT , Rothstein HR . Basics of meta‐analysis: I(2) is not an absolute measure of heterogeneity. Res Synth Methods. 2017;8(1):5‐18. doi:10.1002/jrsm.1230 28058794

[29] Borenstein M , Hedges LV , Higgins JPT , Rothstein HR . Prediction intervals. Introduction to Meta‐Analysis; 2009:127‐133.

[30] Bakr AF , Al‐Abd M , Karkour T . Fetal pulse oximetry and neonatal outcome: a study in a developing country. J Perinatol. 2005;25:759‐762. doi:10.1038/sj.jp.7211406 16281048

[31] Biringer K , Danko J , Žúbor P , et al. Biophysical methods in diagnosis of intrapartal fetal hypoxia. Cesk Gynekol. 2011;76:222‐229. 21838154

[32] Carbonne B , Cudeville C , Sivan H , et al. Fetal oxygen saturation measured by pulse oximetry during labour with clear or meconium‐stained amniotic fluid. Eur J Obstet Gynecol Reprod Biol. 1997;72(Suppl):S51‐S55. doi:10.1016/s0301-2115(97)02718-8 9134413

[33] Kühnert M , Seelbach‐Göebel B , Butterwegge M . Predictive agreement between the fetal arterial oxygen saturation and fetal scalp pH: results of the German multicenter study. Am J Obstet Gynecol. 1998;178(2):330‐335. doi:10.1016/s0002-9378(98)80021-5 9500495

[34] Nikolov A , Dimitrov A , Iarukova N , et al. Intrapartum oxygen saturation in fetus with symptoms of distress shown during fetal cardiotocograph monitoring. Akush Ginekol (Mosk). 2004;43:3‐10. 15518277

[35] Nonnenmacher A , Hopp H , Dudenhausen J . Predictive value of pulse oximetry for the development of fetal acidosis. J Perinat Med. 2010;38(1):83‐86. doi:10.1515/JPM.2010.006 19954413

[36] Rijnders RJ , Mol BW , Reuwer PJ , et al. Is the correlation between fetal oxygen saturation and blood pH sufficient for the use of fetal pulse oximetry? J Matern Fetal Neonatal Med. 2002;11(2):80‐83. doi:10.1080/jmf.11.2.80.83 12375547

[37] Vardon D , Hors Y , Grossetti E , Creveuil C , Herlicoviez M , Dreyfus M . Fetal pulse oximetry: Clinical practice. J Gynecol Obstet Biol Reprod (Paris). 2008;37:697‐704. doi:10.1016/j.jgyn.2008.05.004 18614298

[38] Vitoratos N , Salamalekis E , Saloum J , Makrakis E , Creatsas G . Abnormal fetal heart rate patterns during the active phase of labor: the value of fetal oxygen saturation. J Matern Fetal Neonatal Med. 2002;11(1):46‐49. doi:10.1080/jmf.11.1.46.49 12380608

[39] Bloom SL , Swindle RG , McIntire DD , Leveno KJ . Fetal pulse oximetry: duration of desaturation and intrapartum outcome. Obstet Gynecol. 1999;93(6):1036‐1040. doi:10.1016/S0029-7844(98)00565-1 10362177

[40] Csitári IK , Pasztuhov A , László A . The reliability of fetal pulse oximetry: the effect of fetal oxygen saturation below 30% on perinatal outcome. Eur J Obstet Gynecol Reprod Biol. 2008;136(2):160‐164. doi:10.1016/j.ejogrb.2007.02.021 17467877

[41] East CE , Dunster KR , Colditz PB , Nath CE , Earl JW . Fetal oxygen saturation monitoring in labour: an analysis of 118 cases. Aust N Z J Obstet Gynaecol. 1997;37:397‐401. doi:10.1111/j.1479-828X.1997.tb02446.x 9429700

[42] Grignaffini A , Soncini E , Ronzoni E , et al. Meconium‐stained amniotic fluid and fetal oxygen saturation measured by pulse oximetry during labour. Acta Biomed Ateneo Parmense. 2004;75:45‐52. 15301290

[43] Langer B , Boudier E , Haddad J , Pain L , Schlaeder G . Fetal pulse oximetry during labor of 62 patients. Fetal Diagn Ther. 1996;11:37‐45. doi:10.1159/000264277 8719720

[44] Linhartova L , Kurtansky A , Suska P . Correlation between fetal blood oxygen saturation and umbilical blood pH values. Bratisl Med J. 2009;110:684‐687. 20120434

[45] Salamalekis E , Vitoratos N , Loghis C , Panayotopoulos N , Kassanos D , Creatsas G . Evaluation of fetal heart rate patterns during the second stage of labor through fetal oximetry. Gynecol Obstet Investig. 1999;48:151‐154. doi:10.1159/000010162 10545735

[46] Siristatidis C , Salamalekis E , Kassanos D , Loghis C , Creatsas G . Evaluation of fetal intrapartum hypoxia by middle cerebral and umbilical artery Doppler velocimetry with simultaneous cardiotocography and pulse oximetry. Arch Gynecol Obstet. 2004;270(4):265‐270. doi:10.1007/s00404-003-0556-z 14600768

[47] Stiller R , Mering RV , König V , von Mering R , Huch A , Huch R . How well does reflectance pulse oximetry reflect intrapartum fetal acidosis? Am J Obstet Gynecol. 2002;186(6):1351‐1357. doi:10.1067/mob.2002.122411 12066121

[48] McNamara H , Chung DC , Lilford R , Johnson N . Do fetal pulse oximetry readings at delivery correlate with cord blood oxygenation and acidaemia? Br J Obstet Gynaecol. 1992;99:735‐738. 1420012 10.1111/j.1471-0528.1992.tb13874.x

[49] Carbonne B , Audibert F , Segard L , Sebban E , Cabrol D , Papiernik E . Fetal pulse oximetry: correlation between changes in oxygen saturation and neonatal outcome. Preliminary report on 39 cases. Eur J Obstet Gynecol Reprod Biol. 1994;57:73‐77. Preliminary report on 39 cases. 7859908 10.1016/0028-2243(94)90046-9

[50] Carbonne B , Langer B , Goffinet F , et al. Multicenter study on the clinical value of fetal pulse oximetry: II. Compared predictive values of pulse oximetry and fetal blood analysis. Am J Obstet Gynecol. 1997;177:593‐598. doi:10.1016/S0002-9378(97)70151-0 9322629

[51] Bloom SL , Spong CY , Thom E , et al. Fetal pulse oximetry and cesarean delivery. N Engl J Med. 2006;355:2195‐2202. doi:10.1056/NEJMoa061170 17124017

[52] Caliskan E , Cakiroglu Y , Corakci A , Ozeren S . Reduction in caesarean delivery with fetal heart rate monitoring and intermittent pulse oximetry after induction of labour with misoprostol. J Matern Fetal Neonatal Med. 2009;22(5):445‐451. doi:10.1080/14767050802613207 19530004

[53] Çalişkan E , Doǧer E , Çakiroǧlu Y , et al. The effect of fetal pulse oximetry on neonatal outcomes of fetuses with the diagnosis of intrauterine growth restriction. Turk Jinekol Obstet Dernegi Derg. 2009;6:35‐40.

[54] East CE , Brennecke SP , King JF , et al. The effect of intrapartum fetal pulse oximetry, in the presence of a nonreassuring fetal heart rate pattern, on operative delivery rates: a multicenter, randomized, controlled trial (the FOREMOST trial). Am J Obstet Gynecol. 2006;194:606.e601‐606.e616. doi:10.1016/j.ajog.2005.08.051 16522387

[55] Fernández Andrés I , Martínez Montero I . Fetal pulse oximetry. Intrapartum foetal hypoxia evaluation. Comparative study with invasive techniques concerning foetal welfare. An Sist Sanit Navar. 2004;27(2):179‐189. doi:10.4321/s1137-66272004000300003 15381950

[56] Garite TJ , Dildy GA , McNamara H , et al. A multicenter controlled trial of fetal pulse oximetry in the intrapartum treatment of nonreassuring fetal heart rate patterns. Am J Obstet Gynecol. 2000;183:1049‐1058. 11084540 10.1067/mob.2000.110632

[57] Klauser CK , Christensen EE , Chauhan SP , et al. Use of fetal pulse oximetry among high‐risk women in labour: a randomized clinical trial. Am J Obstet Gynecol. 2005;192:1810‐1819. 15970816 10.1016/j.ajog.2004.12.047

[58] Kuhnert M , Schmidt S . Intrapartum management of nonreassuring fetal heart rate patterns: a randomized controlled trial of fetal pulse oximetry. Am J Obstet Gynecol. 2004;191(6):1989‐1995. doi:10.1016/j.ajog.2004.04.036 15592281

[59] Valverde M , Puertas AM , Lopez‐Gallego MF , Carrillo MP , Aguilar MT , Montoya F . Effectiveness of pulse oximetry versus fetal electrocardiography for the intrapartum evaluation of nonreassuring fetal heart rate. Eur J Obstet Gynecol Reprod Biol. 2011;159(2):333‐337. doi:10.1016/j.ejogrb.2011.09.021 21978943

[60] East CE , Brennecke SP , Chan FY , King JF , Beller EM , Colditz PB . Clinicians' evaluations of fetal oximetry sensor placement in a multicentre randomised trial (the FOREMOST trial). Aust N Z J Obstet Gynaecol. 2006;46:234‐239. doi:10.1111/j.1479-828X.2006.00568.x 16704479

[61] Nellcor Puritan Bennett . N‐400 Fetal Pulse Oximetry Monitor Technical Manual. Accessed April 29, 2025. http://www.frankshospitalworkshop.com/equipment/documents/pulse_oximeter/service_manuals/Nellcor_N‐400_Pulse_Oximeter_‐_Service_manual.pdf

[62] Luttkus AK , Stupin JH , Callsen TA , Dudenhausen JW . Feasibility of simultaneous application of fetal electrocardiography and fetal pulse oximetry. Acta Obstet Gynecol Scand. 2003;82:443‐448. doi:10.1034/j.1600-0412.2003.00134.x 12752075

[63] Shamseer L , Moher D , Clarke M , et al. Preferred reporting items for systematic review and meta‐analysis protocols (PRISMA‐P) 2015: elaboration and explanation. BMJ. 2015;349:g7647. 10.1136/bmj.g7647 25555855

[64] Sobotková D , Kučerová I , Dittrichová J , et al. Psychomotor development of children with signs of intrapartum hypoxia and monitored by intrapartum fetal pulse oxymetry. Cesk Gynekol. 2004;69:114‐120. 15748038

