# Supplementary material for: Association between intrapartum fetal pulse oximetry and adverse perinatal and long‐term outcomes: A systematic review and meta‐analysis
Source: Int J Gynaecol Obstet. 2025 Jun 9;170(3):1038–47. doi: 10.1002/ijgo.70242 (PMC12374029; doi:10.1002/ijgo.70242)
Supplement: Supplementary file 1 — Appendix S1. Search strategy. Appendix S2. Moose checklist for meta‐analyses of observational studies. Appendix S3. PRISMA 2020 checklist. Appendix S4. PRISMA diagram. Appendix S5. Details of included studies. Appendix S6. Correlation between fetal oxygen saturation and umbilical cord pH values. Appendix S7. Additional data. Appendix S8. Quality assessment and GRADE analysis. Appendix S9. Analyses. Appendix S10. Prediction intervals. [file IJGO-170-1038-s001.docx]

**Association between Intrapartum Fetal Pulse Oximetry and Adverse Perinatal and Long-term Outcomes: a Systematic Review and Meta-analysis Appendix**

Table of Contents

[Appendix 1. Search Strategy 2](#_Toc170916902)

[Appendix 2. Moose Checklist for Meta-Analyses of Observational Studies 8](#_Toc170916903)

[Appendix 3. PRISMA 2020 Checklist 10](#_Toc170916904)

[Appendix 4. PRISMA Diagram 13](#_Toc170916905)

[Appendix 5. Details of included studies 14](#_Toc170916906)

[Appendix 6. Correlation between fetal oxygen saturation and umbilical cord pH values 47](#_Toc170916907)

[Appendix 7: Additional Data 51](#_Toc170916908)

[Appendix 8. Quality Assessment and GRADE Analysis 61](#_Toc170916909)

[Appendix 9. Analyses 80](#_Toc170916910)

[Appendix 10. Prediction Intervals 92](#_Toc170916911)

[References 95](#_Toc170916912)

# Appendix 1. Search Strategy

Table S1. Search Strategy

1. **PubMed**

| **PubMed** | **#1** | (labor, obstetric[MeSH Terms]) OR (fetal monitoring[MeSH Terms]) OR (labour[Title/Abstract]) OR (labor[Title/Abstract]) OR (intra-partum[Title/Abstract]) OR (intrapartum[Title/Abstract]) OR ("intra partum"[Title/Abstract]) OR ("fetal monitoring"[Title/Abstract]) |
| --- | --- | --- |
|  | **#2** | (oximetry[MeSH Terms]) OR (blood gas monitoring, transcutaneous[MeSH Terms]) OR (oxygen saturation[MeSH Terms]) OR (oximetry[Title/Abstract]) OR ("oxygen saturation"[Title/Abstract]) OR ("blood gas monitoring"[Title/Abstract]) OR (Sp02[Title/Abstract]) |
|  | **#3** | #1 AND #2 |

1. **CINAHL (Plus with Full Text)**

| **CINAHL** | **#1** | (MH “Labor+) OR (MH “Intrapartum Care+”) OR (MH “Fetal Monitoring+”) OR TI ( “Fetal Monitoring" OR “foetal monitoring” OR "labour OR labor OR intrapartum OR intra-partum OR “intra partum” ) OR AB ( “Fetal Monitoring" OR “foetal monitoring” OR "labour OR labor OR intrapartum OR intra-partum” |
| --- | --- | --- |
|  | **#2** | (MH "Oximetry+") OR (MH "Pulse Oximetry") OR (MH "Blood Gas Monitoring, Transcutaneous") OR TI ( Oximetry OR "blood gas monitoring" OR "Oxygen Saturation" OR Sp02 ) OR AB ( Oximetry OR "blood gas monitoring" OR "Oxygen Saturation" OR Sp02 ) |
|  | **#3** | #1 AND #2 |

1. **EMBASE**

| **EMBASE** | **#1** | 'labor'/exp (EMTREE) OR 'intrapartum care'/exp (EMTREE) OR 'fetus monitoring'/exp  OR 'fetus monitoring':ab,ti OR 'fetal monitoring':ab,ti OR 'foetal monitoring':ab,ti OR labour:ab,ti OR labor:ab,ti OR intrapartum:ab,ti OR 'intra partum':ab,ti |
| --- | --- | --- |
|  | **#2** | 'oximetry'/exp OR 'pulse oximetry'/exp OR 'oxygen saturation'/exp OR 'transcutaneous oxygen monitoring'/exp OR 'transcutaneous oxygen monitoring':ab,ti OR oximetry:ab,ti OR 'blood gas monitoring':ab,ti OR sp02:ab,ti OR 'oxygen saturation':ab,ti |
|  | **#3** | #1 AND #2 |

1. **Web of Science**

| **Web of Science** | **#1** | (TS=("Fetal Monitoring" OR labor OR labour OR intrapartum OR intra-partum OR "intra partum")) |
| --- | --- | --- |
|  | **#2** | TS=(oximetry OR "pulse oximetry" OR "blood gas monitoring" OR "oxygen saturation") |
|  | **#3** | #1 AND #2 |

1. **The Cochrane Library**

| **The Cochrane Library** | **#1** | "labor, obstetric"[MeSH Terms] OR "Fetal Monitoring"[MeSH Terms] OR "labour"[Title/Abstract/KW] OR "labor"[Title/Abstract/KW] OR "intrapartum"[Title/Abstract/KW] OR "intra-partum"[Title/Abstract/KW] |
| --- | --- | --- |
|  | **#2** | "Oximetry"[MeSH Terms] OR "Oxygen Saturation"[MeSH Terms] OR "blood gas monitoring, transcutaneous"[MeSH Terms] OR Oximetry[Title/Abstract/KW] OR "blood gas monitoring"[Title/Abstract/KW] OR "Oxygen Saturation"[Title/Abstract/KW] OR Sp02[Title/Abstract/KW] |
|  | #3 | #1 and #2 |

1. **ClinicalTrials.Gov**

| **ClinicalTrials.Gov** | **#1** | “Fetal Monitoring" OR “foetal monitoring” OR "labour OR labor OR intrapartum OR intra-partum |
| --- | --- | --- |
|  | **#2** | Oximetry OR "blood gas monitoring" OR "Oxygen Saturation" OR Sp02 |
|  | #3 | #1 and #2 |

1. **WHO ICTRP**

| **WHO ICTRP** | #1 | Fetal Monitoring OR foetal monitoring OR labour OR labor OR intrapartum OR intra-partum |
| --- | --- | --- |
|  | #2 | Oximetry OR blood gas monitoring OR Oxygen Saturation |
|  | #3 | #1 and #2 |

# Appendix 2. Moose Checklist for Meta-Analyses of Observational Studies

| **Item No** | **Recommendation** | **Reported on Page No** |
| --- | --- | --- |
| Reporting of background should include | | |
| 1 | Problem definition | 6 |
| 2 | Hypothesis statement | 7 |
| 3 | Description of study outcome(s) | 8 |
| 4 | Type of exposure or intervention used | 7 |
| 5 | Type of study designs used | 8 |
| 6 | Study population | 7 |
| Reporting of search strategy should include | | |
| 7 | Qualifications of searchers (e.g., librarians and investigators) | 9 |
| 8 | Search strategy, including time period included in the synthesis and key words | 8,9 Appendix 1 |
| 9 | Effort to include all available studies, including contact with authors | 10 |
| 10 | Databases and registries searched | 10 |
| 11 | Search software used, name and version, including special features used (e.g., explosion) | 9 |
| 12 | Use of hand searching (e.g., reference lists of obtained articles) | 9 |
| 13 | List of citations located and those excluded, including justification | 9, Appendix 1 |
| 14 | Method of addressing articles published in languages other than English | 9 |
| 15 | Method of handling abstracts and unpublished studies | 8, 9 |
| 16 | Description of any contact with authors | 9 |
| Reporting of methods should include | | |
| 17 | Description of relevance or appropriateness of studies assembled for assessing the hypothesis to be tested | 9 |
| 18 | Rationale for the selection and coding of data (e.g., sound clinical principles or convenience) | 9, protocol |
| 19 | Documentation of how data were classified and coded (e.g., multiple raters, blinding and interrater reliability) | 9 |
| 20 | Assessment of confounding (e.g., comparability of cases and controls in studies where appropriate) | 10 |
| 21 | Assessment of study quality, including blinding of quality assessors, stratification or regression on possible predictors of study results | 10 |
| 22 | Assessment of heterogeneity | 10 |
| 23 | Description of statistical methods (e.g., complete description of fixed or random effects models, justification of whether the chosen models account for predictors of study results, dose-response models, or cumulative meta-analysis) in sufficient detail to be replicated | 10 |
| 24 | Provision of appropriate tables and graphics | Tables and figures in manuscript and Appendices |
| Reporting of results should include | | |
| 25 | Graphic summarizing individual study estimates and overall estimate | Figures 1-4 in manuscript and Appendices 7 and 9 |
| 26 | Table giving descriptive information for each study included | Appendix 5 |
| 27 | Results of sensitivity testing (e.g., subgroup analysis) | 16, 17, Appendix 9 |
| 28 | Indication of statistical uncertainty of findings | 17, Appendix 10 |

# Appendix 3. PRISMA 2020 Checklist

| **Section and Topic** | **Item #** | **Checklist item** | **Location where item is reported** |
| --- | --- | --- | --- |
| **TITLE** | | |  |
| Title | 1 | Identify the report as a systematic review. | 1 |
| **ABSTRACT** | | |  |
| Abstract | 2 | See the PRISMA 2020 for Abstracts checklist. | 4 |
| **INTRODUCTION** | | |  |
| Rationale | 3 | Describe the rationale for the review in the context of existing knowledge. | 6 |
| Objectives | 4 | Provide an explicit statement of the objective(s) or question(s) the review addresses. | 7 |
| **METHODS** | | |  |
| Eligibility criteria | 5 | Specify the inclusion and exclusion criteria for the review and how studies were grouped for the syntheses. | 7,8 |
| Information sources | 6 | Specify all databases, registers, websites, organisations, reference lists and other sources searched or consulted to identify studies. Specify the date when each source was last searched or consulted. | 9, Appendix 1 |
| Search strategy | 7 | Present the full search strategies for all databases, registers and websites, including any filters and limits used. | Appendix 1 |
| Selection process | 8 | Specify the methods used to decide whether a study met the inclusion criteria of the review, including how many reviewers screened each record and each report retrieved, whether they worked independently, and if applicable, details of automation tools used in the process. | 9 |
| Data collection process | 9 | Specify the methods used to collect data from reports, including how many reviewers collected data from each report, whether they worked independently, any processes for obtaining or confirming data from study investigators, and if applicable, details of automation tools used in the process. | 9 |
| Data items | 10a | List and define all outcomes for which data were sought. Specify whether all results that were compatible with each outcome domain in each study were sought (e.g. for all measures, time points, analyses), and if not, the methods used to decide which results to collect. | 8 and protocol |
|  | 10b | List and define all other variables for which data were sought (e.g. participant and intervention characteristics, funding sources). Describe any assumptions made about any missing or unclear information. | 8 and protocol |
| Study risk of bias assessment | 11 | Specify the methods used to assess risk of bias in the included studies, including details of the tool(s) used, how many reviewers assessed each study and whether they worked independently, and if applicable, details of automation tools used in the process. | 9,10 |
| Effect measures | 12 | Specify for each outcome the effect measure(s) (e.g. risk ratio, mean difference) used in the synthesis or presentation of results. | 10 |
| Synthesis methods | 13a | Describe the processes used to decide which studies were eligible for each synthesis (e.g. tabulating the study intervention characteristics and comparing against the planned groups for each synthesis (item #5)). | 10 |
|  | 13b | Describe any methods required to prepare the data for presentation or synthesis, such as handling of missing summary statistics, or data conversions. | 10 |
|  | 13c | Describe any methods used to tabulate or visually display results of individual studies and syntheses. | 10 |
|  | 13d | Describe any methods used to synthesize results and provide a rationale for the choice(s). If meta-analysis was performed, describe the model(s), method(s) to identify the presence and extent of statistical heterogeneity, and software package(s) used. | 10 |
|  | 13e | Describe any methods used to explore possible causes of heterogeneity among study results (e.g. subgroup analysis, meta-regression). | 10, 11 |
|  | 13f | Describe any sensitivity analyses conducted to assess robustness of the synthesized results. | 10, 11 |
| Reporting bias assessment | 14 | Describe any methods used to assess risk of bias due to missing results in a synthesis (arising from reporting biases). | 10 |
| Certainty assessment | 15 | Describe any methods used to assess certainty (or confidence) in the body of evidence for an outcome. | 10 |
| **RESULTS** | | |  |
| Study selection | 16a | Describe the results of the search and selection process, from the number of records identified in the search to the number of studies included in the review, ideally using a flow diagram. | 11, Appendix 1 |
|  | 16b | Cite studies that might appear to meet the inclusion criteria, but which were excluded, and explain why they were excluded. | Appendix 1 |
| Study characteristics | 17 | Cite each included study and present its characteristics. | Appendix 5 |
| Risk of bias in studies | 18 | Present assessments of risk of bias for each included study. | Appendix 8 |
| Results of individual studies | 19 | For all outcomes, present, for each study: (a) summary statistics for each group (where appropriate) and (b) an effect estimates and its precision (e.g. confidence/credible interval), ideally using structured tables or plots. | Figures 1-4, Appendix 7 |
| Results of syntheses | 20a | For each synthesis, briefly summarise the characteristics and risk of bias among contributing studies. | 16, Appendix 8 |
|  | 20b | Present results of all statistical syntheses conducted. If meta-analysis was done, present for each the summary estimate and its precision (e.g. confidence/credible interval) and measures of statistical heterogeneity. If comparing groups, describe the direction of the effect. | 11-15  Appendix 6,7,9 |
|  | 20c | Present results of all investigations of possible causes of heterogeneity among study results. | 16,17, Appendix 9 |
|  | 20d | Present results of all sensitivity analyses conducted to assess the robustness of the synthesized results. | 16, 17, Appendix 9 |
| Reporting biases | 21 | Present assessments of risk of bias due to missing results (arising from reporting biases) for each synthesis assessed. | 17, Appendix 7 |
| Certainty of evidence | 22 | Present assessments of certainty (or confidence) in the body of evidence for each outcome assessed. | 17, Appendix 8 |
| **DISCUSSION** | | |  |
| Discussion | 23a | Provide a general interpretation of the results in the context of other evidence. | 17, 18 |
|  | 23b | Discuss any limitations of the evidence included in the review. | 19 |
|  | 23c | Discuss any limitations of the review processes used. | 19 |
|  | 23d | Discuss implications of the results for practice, policy, and future research. | 18 |
| **OTHER INFORMATION** | | |  |
| Registration and protocol | 24a | Provide registration information for the review, including register name and registration number, or state that the review was not registered. | 2, 9 |
|  | 24b | Indicate where the review protocol can be accessed, or state that a protocol was not prepared. | 2, 9 |
|  | 24c | Describe and explain any amendments to information provided at registration or in the protocol. | 19, Described in published protocol and PROSPERO |
| Support | 25 | Describe sources of financial or non-financial support for the review, and the role of the funders or sponsors in the review. | 5 |
| Competing interests | 26 | Declare any competing interests of review authors. | 2 |
| Availability of data, code and other materials | 27 | Report which of the following are publicly available and where they can be found: template data collection forms; data extracted from included studies; data used for all analyses; analytic code; any other materials used in the review. | Available on request |

# Appendix 4. PRISMA Diagram

References from databases/registers **(n = 3280)** (as **n = 3277** studies)

Embase (n = 1465)

PubMed (n = 711)

Web of Science (n = 606)

Cochrane Library (n = 221)

CINAHL (n = 199)

ClinicalTrials.gov (n=64)

WHO ICTRP (n=14)

**Identification**

Studies included in review **(n = 47)**

Studies excluded **(n = 1829)**

Studies not retrieved **(n = 4)**

Studies assessed for eligibility **(n = 205)**

Studies sought for retrieval **(n = 209)**

Studies screened **(n = 2042)**

Studies excluded **(n = 162)**

Wrong outcomes (n = 70)

Wrong comparator (n = 5)

Wrong intervention (n = 70)

Only abstract available (n = 2)

Wrong patient population (n = 11)

References removed **(n = 1235)**

Duplicates identified manually (n = 64)

Duplicates identified by Covidence (n = 1169)

**Screening**

**Included**

Figure 1. PRISMA Diagram

# Appendix 5. Details of included studies

Table 1. Randomised Control Trials of Fetal Pulse Oximetry

| **Study ID** | **Multi-centre or Single-centre** | **Total participants** | **Country** | **Participant Characteristics** | **Fetal oxygen saturation cut off value** | **Comparison Groups** | **Type of FHR** | **Outcomes** | **Signal %** |
| --- | --- | --- | --- | --- | --- | --- | --- | --- | --- |
| **Bloom 2006^1^** | Multi-centre | 5341 | United States of America | Nulliparous women from 36 weeks’ gestation in early or active labour. Reassuring and NRFHR patterns | <30% for at least 2 consecutive minutes: left to the discretion of the attending physician | FHR alone versus FHR + FSp02 | Electronic FHR monitoring and intrauterine pressure catheter | CS rates.  CS for NRFS/dystocia  Neonatal morbidity.  UA pH<7.0.  5 minute Apgar score <4  NICU admission  Neonatal intubation,  HIE  Neonatal death | 75.2% in open group/ 72.9% in masked group |
| **Caliskan 2009 I^2^** | Single centre | 230 | Turkey | ≥34 weeks undergoing induction of labour with oral misoprostol with a bishops score ≤5. RFS monitoring at enrolment. | <30 for 3 minutes: intrauterine resuscitative measures  <30 for 10 mins: delivery expedited | FHR alone versus FHR + FSp02 | Electronic FHR monitoring | CS rates, CS for NRFS/dystocia, UA pH≤7.15, UA BE < -10, neonatal intubation, NICU admission, 5 minute Apgar <7, | Not reported |
| **Caliskan 2009 II^3^** | Single-centre | 91 | Turkey | ≥35 weeks with a pregnancy complicated by IUGR undergoing induction of labour with oral misoprostol. | Measured for 10 minutes in 30 minute intervals. Measured continuously in the setting of a NRFHR pattern. ≤30% for 2 minutes: expedite delivery. | FHR alone versus FHR + FSp02 | Electronic FHR monitoring | CS rates, CS for NRFS/dystocia UA pH <7.15, UA BE <-8, 5 minute Apgar score <7 | Not reported |
| **East 2006^4^** | Multi-centre | 600 | Australia | ≥36 weeks with NRFHR patterns in early or active labour. | <30% for 10 minutes or unavailable: intrauterine resuscitative measures, FBS or expedite delivery | FHR alone versus FHR + FSp02 | Electronic FHR monitoring (CTG) | Operative delivery rates. Operative delivery for NRFS. Operative delivery for dystocia. UA pH <7.15 and <7.00. UA BE< -10 and <-16. UA lactate levels. 5 minute Apgar<7. NICU admissions, CPR within 24 hours of life | Median: 83% (IQR 68%, 91%) |
| **Garite 2000^5^** | Multi-centre | 1010 | United States of America | ≥36 weeks’ gestation with NRFHR patterns in early or active labour. | <30% for the entire duration between two uterine contractions or un intrauterine resuscitation before expediting delivery | FHR alone versus FHR + FSp02 | Electronic FHR monitoring (CTG) | OD rates, OD for NRFS/dystocia. UA BE≤-10, UA pH <7.0/7.05/7.10. 5 minute Apgar<7, NICU admission, Neonatal intubation, neonatal death | 67% |
| **Klauser 2005^6^** | Single-centre | 327 | United States of America | ≥ 28 weeks’ gestation with NRFHR patterns in early or active labour | < 30% of 3 minutes: intrauterine resuscitation before expediting delivery | FHR alone versus FHR + FSp02 | Electronic FHR monitoring | CS rates. CS for NRFS/dystocia. UA pH < 7.0/7.10. 5 minute Apgar < 7, NICU admission. | Not reported |
| **Kuhnert 2004^7^** | Single-centre | 146 | Germany | ≥ 36 weeks in early or active labour with NRFHR patterns | < 30% for 10 minutes or repeatedly < 30%: Follow up FBS | FHR + FBS versus FHR + FBS + FSp02 | Electronic FHR monitoring (CTG) | OD rates. OD for NRFS/dystocia. UA pH< 7.20/< 7.10. Neonatal death. Correlation between FSp02 and UA pH and fetal scalp pH. | 98.5% |
| **Valverde 2011^8^** | Single-centre | 180 | Spain | ≥37 weeks with NRFHR patterns in the second stage of labour | 10-30%: FBS and scalp stimulation. < 30%: expedite delivery | FHR + fetal ECG versus FHR + FSp02 | Electronic FHR monitoring (CTG) | OD rates. OD for NRFS/dystocia. UA pH and BE levels. UV pH levels. 5 minute Apgar score <6. | 49.92% |

**Key:** BE = Base excess, CPR = cardiopulmonary resuscitation, CS = Cesarean section, FBS = Fetal blood sample, FHR = Fetal heart rate, FspO2 = Fetal oxygen saturation, HIE = hypoxic ischaemic encephalopathy, IUGR = intrauterine growth restriction, NICU = neonatal intensive care unit, NRFHR = Non-reassuring fetal heart rate, NRFS = Non-reassuring fetal status, OD = operative delivery, RFS = Reassuring fetal status, UA = umbilical artery, UV = umbilical vein

Table 2. Non-Randomised Studies of Fetal Pulse Oximetry

| **Study ID** | **Study Type** | **Total participants** | **Country** | **Participant Characteristics** | **Fetal Oxygen Saturation Cut Off Values** | **Did FSp02 influence clinical management** | **Comparison Groups** | **Type of FHR monitoring** | **Outcomes** | **Signal Quality % (mean unless otherwise stated)** |
| --- | --- | --- | --- | --- | --- | --- | --- | --- | --- | --- |
| **Bakr 2005^9^** | Cohort | 150 | Egypt | Women in labour with NRFHR pattern | Average FSp02 over a 30 minute period ≤30% and ≤40% | No | FSp02 ≤ 30% versus >30%. FSp02 ≤40% versus >40% | Electronic FHR monitoring | UA pH ≤ 7.15. Fetal scalp pH ≤7.20. Abnormal neonatal outcome (composite) | Not reported |
| **Biringer 2011^10^** | Case Control | 67 | Slovakia | ≥ 37 weeks in labour with RFHR or NRFHR pattern. | < 30% for more than 10 minutes | No | UA pH <7.15 versus UA pH > 7.15 in order to assess FPO, fetal ECG and CTG as fetal monitoring methods | Electronic FHR monitoring (CTG) | Number of cases of FSP02<30% among UA pH < 7.15 group. How accurately a UA pH<7.15 can predict FSp02 <30%, an abnormal fetal ECG and a NRFHR pattern | Not reported |
| **Bloom 1999^11^** | Cohort | 129 | United States of America | ≥36 weeks’ gestation with uncomplicated pregnancies in labour with reassuring and NRFHR patterns | <30% for 10 seconds. Investigating increasing duration of low FSp02 up to 9 minutes | No | FSp02<30% versus FSp02 ≥ 30% | Electronic FHR monitoring (CTG) | CS rates. CS for NRFS/dystocia. UA pH ≤7.20. 5 minute Apgar score ≤3. Composite outcome for potential fetal compromise | Not reported |
| **Butterwegge 1997^12^** | Cohort | 200 | Germany | > 32 weeks’ gestation with NRFHR pattern in labour. | FSp02 values were measured during an FBS over a period of 5-10 minutes. Mean FSp02 levels were calculated at 4-7cm, 8-10cm and in the second stage of labour | No | Correlation between FSp02 and FHR and scalp pH. | Electronic FHR monitoring (CTG) | Correlation between FSp02 and FHR and scalp pH. | 80% |
| **Carbonne 1994^13^** | Cohort | 27 | France | ≥37 weeks’ gestation with NFHR pattern in the first stage of labour. Patients who underwent a Cesarean section were excluded | Average FSp02 during the last 5 minutes of CTG if delay between last FSp02 and birth < 10 minutes. Correlation and FSp02< 40%. FSp02 < 30% (extracted from scatter plot) | No | Correlation between Fsp02 and UV pH<7.20 and 5 minute Apgar score< 5. Predictive values for FSp02 < 40% and UV pH <7.20. FSp02 <40% versus ≥ 40%. FSp02 < 30% versus ≥ 30% | Electronic FHR monitoring (CTG) | UV pH <7.20.  5 minute Apgar score <7 | 1st stage = 72.8 (SD= 17.6%). 2nd stage = 56.4 (SD= 23.8%) |
| **Carbonne 1997^14^** | Cohort | 164 | France | ≥ 36 weeks’ gestation in labour with a NRFHR pattern | Average FSp02 < 30% over 30 minute period before second stage/before CS or during second stage | No | FSp02 < 30% versus ≥ 30%. Correlation between FSp02 in the last 10 minutes before birth and umbilical artery and vein pH | Electronic FHR monitoring (CTG) | CS for NRFS, UA pH ≤ 7.15, 5 minute Apgar score ≤ 7. NICU admission. Neonatal resuscitation. Correlation between FSp02 and UA and UV pH. | 1st stage: 64.7 (SD= 32%) 2nd stage: 54 (SD= 26%) |
| **Chua 1999^15^** | Cohort | 73 | Singapore | Women in labour with reassuring or NRFHR patterns | Mean FSp02 for the last 10 minutes before birth | No | Correlation between FSp02 levels in the 10 minutes before birth and umbilical artery and venous pH and BE. | Electronic FHR monitoring | Correlation between FSp02 levels in the 10 minutes before birth and umbilical artery and venous pH and BE. 5 minute Apgar score < 5 and < 7. | Median 68.95% (range = 3.2-100%) |
| **Csitári 2008^16^** | Non-randomised control trial | 301 | Hungary | ≥ 37 weeks in labour with RFHR and NRFHR patterns | <30% for more than 10 minutes: Cesarean delivery unless vaginal birth imminent | Yes | FSp02 < 30% versus ≥ 30% | Electronic FHR monitoring (CTG) | CS rates. CS for NRFS. CS for dystocia. UA pH < 7.20. UA BE ≤ -14. 5 minute Apgar score <7. NICU admission. Neonatal death | Not reported |
| **East 1997^17^** | Cohort | 118 | Australia | ≥ 36 weeks in labour RFHR and NRFHR patterns | < 30% for entire monitoring period.  Mean FSp02 < 30% for last 10 minutes before birth | No | < 30% FSp02 versus ≤ 30% FSp02 | Electronic FHR monitoring (CTG) | UA pH ≤ 7.20. 5 minute Apgar score < 7. | Median 83% (IQR 68%, 91%) |
| **Fernández Andrés 2004^18^** | Non-randomised control | 160 | Spain | ≥37 weeks with a normal risk pregnancy in labour. RFHR and NRFHR patterns | Normal: >30%  Pre-pathological 20-30% for > 20 minutes. Pathological: <20% or 20-30% for >20 minutes: Expedite delivery if FSp02 <20% or 20-30% for more than 10 minutes | Yes | FHR + FBS versus FHR + FBS + FSp02.  Correlation between FSp02 and UA and fetal scalp pH | Electronic FHR monitoring | CS rates. UA pH <7.20. 5 minute Apgar score < 7.  Correlation between FSp02 and UA and fetal scalp pH | Not reporter |
| **Grignaffini 2004^19^** | Non-randomised control trial | 58 | Italy | ≥ 36 weeks in labour with a NRFHR pattern and/or meconium | Mean FSp02 <30% in the first 30 minutes of application and in the last 30 minutes before birth.  Last available FSp02 value before birth <30% (data extracted from scatter plot) | Yes (Retrospective, not explicitly stated) | FHR + FSp02 versus FSp02 alone in the setting of meconium. FSp02 <30% versus FSp02 ≥30% | Electronic FHR monitoring | OD rates. OD for NRFS/dystocia. UA pH < 7.20/7.10. UA BE ≤-8. 5 minute Apgar score <7. Mean UA pH and BE levels. | Not reported |
| **Hájek 2006^20^** | Cohort | 114 | Czech Republic | Women with NRFHR patterns in labour | Pathological: <30% for more than 10 minutes. Intermediate: 31-34%. Normal: >35% | Diagnostic accuracy of FSp02 <30% predicting UA pH ≤7.20, UA BE ≤-10, UA lactate ≥3.7 and neonatal condition (composite) compared to NRFHR and abnormal fetal ECG. | Not stated. | Electronic FHR monitoring (CTG) | Diagnostic accuracy of FSp02 < 30% predicting UA pH ≤ 7.20, UA BE ≤-10, UA lactate ≥3.7 | Not reported |
| **Kuhnert 1998^21^** | Cohort | 46 | Germany | Term pregnancies with NRFHR in labour | ≤30% for 10 minutes | FSp02<30% versus FSp02 ≥ 30%. Diagnostic accuracy of FSP02 < 30% predicting scalp pH < 7.20 | No | Electronic FHR monitoring (CTG) | Mean UA and UV pH. Mean 5 minute Apgar score. UA pH < 7.20/< 7.15. Scalp pH<7.20. NICU admission. Neonatal CPR. Neonatal mortality. Diagnostic accuracy of FSP02 <30% predicting scalp pH<7.20 | Not reported |
| **Kuhnert 2001^22^** | Cohort | - 1000 intrapartum FSp02 measurements | Germany | Women in labour | ≤30% for 10 minutes | FSP02 ≤ 30% versus >30%. Length of time FSP02≤30%. Sensitivity of FSp02 ≤30% for 10 minutes with registration time ≥80% predicting UA pH <7.20. | Not stated | Electronic FHR monitoring (CTG) | Correlation between FSp02 and pH change between two FBS samples. Sensitivity of FSp02 ≤30% for 10 minutes predicting UA pH <7.20 | Not reported |
| **Langer 1996^23^** | Cohort | 62 | France | 36-42 weeks’ gestation with reassuring and NRFHR patterns in labour | Mean FSp02 <30% during active second stage | FSp02 <30% versus FSp02 ≥30%. Correlation between FSp02 and scalp and UA pH. Sensitivity of FSp02 <30% predicting UA pH <7.20. | No | Electronic FHR monitoring (scalp electrode) | UA pH <7.20/7.10. UV pH <7.20. UA and UV BE ≤-10. 5 minute Apgar score <7. Correlation between FSp02 and scalp and UA pH. | Dilation at 4-6cm corresponds to 63.7% (SD= 24.5%), at 6 to 8cm to 60.9% (SD = 25.5%), and for >8cm to 53.4% (SD = 27.2%). 2^nd^ stage = 33.6% (SD =22.6%). |
| **Leszcyznska-Gorzelak 2002** | Cohort | 28 | Poland | ≤37 weeks’ gestation who achieved a normal vaginal birth. RFHR and NRFHR patterns. | 1^st^ stage: average Fsp02 in 10 minute intervals <30%.  2^nd^ stage: average FSP02 in 1 minute intervals <30% | FSp02 < 30% versus FSp02 ≥ 30%. Correlation between FSP02 and 5 minute Apgar score and UV pH. | Not stated | Electronic FHR monitoring (CTG) | Correlation between FSP02 and 5 minute Apgar score and UV pH.  UV pH <7.10. | 81.04% |
| **Linhartova 2009^24^** | Cohort | 76 | Slovakia | 40-41 weeks with NRFHR patterns | <30% for ≤10 minutes | FSp02 <30% for ≤10 minutes versus ≤30%. Correlation between FSp02 and UA pH | Unclear | Electronic FHR monitoring (CTG) | CS rates, CS for NRFS/ dystocia, UA pH<7.20, Correlation between FSP02 and UA pH | Not reporter |
| **Luttkus 1996^25^** | Cohort | 150 | Germany | Labouring women with a suspicion of fetal hypoxia i.e. NRFHR or meconium | Median and Percentiles of SpO2:  Over the entire observation period.  Over the last 30 minutes before delivery.  Duration of SpO2 Below Critical Thresholds:  For the entire observation time: Minimum 3 minutes. For the last 30 minutes before delivery. Critical thresholds = 10th centile: 35% (whole observation), 34% (last 30 mins). 5th centile: 30% (whole observation), 27% (last 30 mins). 3rd centile: 27% (whole observation), 23% (last 30 mins). | Diagnostic accuracy of FSp02 predicting UA<7.20 | No (blinded) | Electronic FHR monitoring (CTG) | Sensitivity, specificity and predictive values of FSp02 35%, 30%, 27% predicting UA pH<7.20 | Not reported |
| **Luttkus 2002^26^** | Cohort | 170 | Germany | Women in labour with NRFHR patterns | SpO2 medians and percentiles were tracked throughout the observation period and specifically during the last 30 minutes before delivery.  SpO2 levels were below critical thresholds (3rd, 5th, and 10th percentiles) for at least 3 minutes during the entire observation and at least 30 seconds in the last 30 minutes before delivery. | FSp02 <30% versus FSp02 ≥30%. Correlation between FSp02 and scalp pH | No | Electronic FHR monitoring | Acidotic composite variable (UA pH ≤7.16 and UA BE≤-9.4mmol/L, Scalp pH <7.20 (scatter plot), correlation between FSp02 and scalp pH | Range = 31% - 96% |
| **Luttkus 2003^27^** | Cohort | 35 | Germany | >36 weeks in labour with a suspicion of fetal hypoxia (NRFHR/meconium) | Drop of FSp02 of at least 20% from baseline level occurring within 1 min or duration of time FSp02 <30% | Examining the coincidence between FPO and FECG concerning signs of fetal compromise | No | Electronic FHR monitoring | UA lactate ≥5.5 mmol/l, UA pH ≤7.20. UA lactate, UA pH | FPO alone: median = 78%. FPO + fetal ECG = 66% |
| **Markwitz 2000** | Cohort | 26 | Poland | 37-41 weeks in labour. RFHR and NRFHR patterns | Mean FSp02 <30% during the last 30 minutes of labour | Correlation between mean FSP02 in last 30mins of labour and UA and UV pH | No | Electronic FHR monitoring (CTG) | UA and UV pH | 48.5 (SD = 14.6%) |
| **McNamara 1992^28^** | Cohort | 37 | UK | Women with an uncomplicated labour | Mean FSP02 in second stage. <30% taken from scatter plots | Correlation between FSp02 and UV Sp02/UV pH/UA pH/5 min Apgar/UV Sp02 | No | Not stated | UA pH <7.20, <7.10.UV pH, UA Sp02, UV Sp02 (scatter plots), Correlation between FSp02 and UV Sp02/UV pH/UA pH/5 min Apgar/UV Sp02 | Data accepted if signal quality >60% |
| **Nikolov 2004^29^** | Cohort | 62 | Bulgaria | 37-42 weeks in labour with NRFHR pattern | ≤30% reading immediately before a CS or within 15 minutes of VD | FSp02 ≤30% versus FSp02 >30%. | No | Electronic FHR monitoring (CTG) | UA pH≤7.15, 5 min Apgar ≤7, NICU, neonatal intubation, NND, abnormal state of newborn (composite variable) | 1^st^ stage: 86.74%.  2^nd^ stage: 77.07% |
| **Nonnenmacher 2010^30^** | Cohort | 119 | Germany | 36-42 weeks in labour with NRFHR patterns | <30% for ≥10mins during the last 60 mins before delivery (did not have to be consecutive) | FSP02 <  30%≥10mins v <30% for <10mins. Diagnostic accuracy of <  30%≥10mins predicting UA pH<7.15 | No | Electronic FHR monitoring (CTG) | UA<7.15 | Nellcor N400 had adequate signal in 49/65 cases; FetalSat achieved reliable signal in over 90% of cases |
| **Rijnders 2002^31^** | Cohort | 65 enrolled, 25 suitable for analysis | Netherlands | >37 weeks in labour with NRFHR patterns or thick meconium | Mean and lowest FSp02 >3mins for the last 30 mins prior to either FBS or delivery.<30% mean and lowest taken from scatter plots | Correlation between FSp02 and scalp and UA pH | No | Electronic FHR monitoring | UA<7.20/7.15/7.1, Scalp pH <7.2, Correlation between FSP02 and Scalp pH/UA pH | Data accepted if signal quality >60% |
| **Roztocil 2000^32^** | Cohort | 68 | Czech Republic | From 36 weeks in labour with NRFHR patterns | <30% for 10 mins | FSP02 < 30% versus ≥ 30% | Yes | Electronic FHR monitoring (CTG) | Mean UA pH, 5 min Apgar, CS rates | Data accepted if signal quality >50% |
| **Salamalekis 1999^33^** | Cohort | 68 | Greece | From 38 weeks in labour. RFHR and NRFHR patterns | Mean FSp02 ≤ 30% in the second stage | FSP02 ≤ 30% versus > 30%. Correlation between FSP02 and UA pH, 5 min Apgar | No | Electronic FHR monitoring (CTG) | Correlation between FSP02 and UA pH, 5 min Apgar (divided into normal and abnormal CTG), UA pH<7.20, 5 min Apgar<7 | 65.4 (SD = 13.9%) |
| **Seelbach-Gobel 1994^34^** | Cohort | 122 | Germany | From 37 weeks in labour with RFHR patterns and NRFHR patterns | <30% for ≥ 10 mins versus < 30% for < 10 mins, correlation | FSP02 < 30% versus ≥ 30%. FSP02 < 30% for < 10mins versus ≥ 10 mins. Correlation between FSp02 and UA/UV/scalp pH | No | Electronic FHR monitoring (CTG) | Correlation between FSp02 and UA/UV/scalp pH - divided by duration of low FSp02, UA pH, UV pH <7.20, | Range = 60-70% |
| **Seelbach-Gobel 1999^35^** | Cohort | 400 | Germany | Normal risk pregnancies between 37 and 42 weeks with RFHR patterns and NRFHR patterns | Duration in minutes of low (≤30%), medium  (>30%-60%), and high (>60%) FSp02. | Duration in minutes of low (≤30%), medium  (>30%-60%), and high (>60%) FSp02 and neonatal outcomes | No | Electronic FHR monitoring (CTG) | UA pH<7.15, BE<-12 mmol/L, diagnostic accuracy of duration  of low FSp02 to predict a decline of scalp pH by >0.05 | 72% (Range = 22-100%) Data only included if signal loss of <30% |
| **Seelbach-Gobel 2005^36^** | Cohort | 136 | Germany | Women from 36-42 weeks with NRFHR patterns | Duration in minutes of low (≤30%), medium  (>30%-60%), and high (>60%) FSp02. <30% for ≥10 mins taken from scatter plots | Duration in minutes of low (≤30%), medium  (>30%-60%), and high (>60%) FSp02 and neonatal outcomes | No | Electronic FHR monitoring (CTG) | Diagnostic accuracy of FSp02 ≤30% for ≥10 or 15 mins predicting scalp/UA pH <7.1/7.15/7.2 and BE ≤-12/-8/-4 mmol/l and scalp pH drop of ≥0.05 and 0.1pH units and BE drop of ≥4mmol/l | Data excluded if signal loss >20% |
| **Siristatidis 2003^37^** | Cohort | 48 | Greece | Group I: Pregnancies affected by IUGR, 33-37 weeks. Group II: Pregnancies not affected by IUGR, 38-40 weeks  Both groups: primiparous women with NRFHR patterns | <30% FSp02 x 2 minutes during the first stage of labour | FSp02 <30% versus FSp02 ≥30% | Yes | Electronic FHR monitoring (CTG) | CS rates. CS for dystocia, CS for NRFS. Mean UA pH, BE, 5min Apgar, | 1st stage: 90 (SD = 4.2%) 2nd stage 92 (SD =6.2%) |
| **Siristatidis 2004^38^** | Cohort | 92 | Greece | Normal risk pregnancies from 38-41 weeks with RFHR and NRFHR patterns | Group A: RFHR, FSP02 >40%  Group B: NRFHR, FSp02 ≥30%  Group C: NRFHR, FSp02 <30% for up to 2 minutes. FSp02  <30% for 2 minutes: EMCS  All groups: first stage of labour | FSP02 <30% versus FSp02 ≥30% | Yes | Electronic FHR monitoring (CTG) | 5 minute Apgar score, neonatal intubation, admission to SCBU, mean UA pH and BE, UA pH <7.20, 5 minute Apgar score <7 | 96% |
| **Skoczylas 2003^39^** | Cohort | 62 | Poland | Women in labour from 37 weeks with NRFHR and RFHR patterns | <30% in late first stage of labour for 30mins | FSP02 <30% versus FSp02 ≥30% | Unclear | Electronic FHR monitoring (CTG) | Diagnostic accuracy of FSp02 < 30% predicting postnatal acidosis (composite variable UA pH <7.20 and/or BE >10 mM/L | Not reported |
| **Sobotkova 2004^40, 41^** | Cohort | 88 | Czech Republic |  | Group I: NRCTG+FPO  Group II: NRCTG alone  Group III: No signs of hypoxia  FPO cut-off value not mentioned | FHR alone versus FHR + FSp02 | Yes | Electronic FHR monitoring (CTG) | Mental, Motor development, Behavioural score, 5 min Apgar scores | Not reported |
| **Stiller 2002^42^** | Cohort | 107 | Switzerland | Women in labour from 36 weeks. RFHR and NRFHR patterns | Mean FSP02 for the total monitoring period/1st stage/2nd stage/final 30 mins before birth. Thresholds of 33 to 36% depending on stage and UA parameters. In the scatter plots we set the cut-off as <30% | Low versus Normal FSP02 | Unclear (don’t think so) | Electronic FHR monitoring (CTG) | UA pH < 7.20/ <7.10, UA BE ≤ -10 (1st stage, 2nd stage, final stage),Diagnostic accuracy of FSP02 20%-36.2% predicting UA pH ≤7.14 and UA BE≤-6.13mmol/L | Overall: 65 (SD = 15.6%). 1st stage = 72.6 (SD= 15.9%) 2nd stage=54.9 (SD = 9.9%)Final stage 44.4 (SD =17.9%) |
| **Tomialowicz 2007^43^** | Cohort | 21 | Poland | Women from 38-42 weeks in the second stage of labour. RFHR and NRFHR patterns | Mean FSp02 during the last 30 minutes of labour | Correlation between FSp02 and neonatal outcome | No | Electronic FHR monitoring (CTG) | Correlation between FSP02 and Apgar score, UV BE, pH, UA pH/BE. | 59.9 (SD = 19.3%) |
| **Uchida 2015^44^** | Cohort | 30 | Japan | Women with a term, uncomplicated pregnancy in the second stage of labour | Mean fetal tissue oxygen saturation in the second stage of labour.  < 30% from scatter plots. | Correlation between fetal tissue oximetry and neonatal outcomes | No | Not stated | Correlation between mean Ft02 and UA pH. UA pH <7.20/7.10 (scatter plots) | 100% during the initial 1 min, 63.3% during the sequential 3 min, 46.7% during the entire 5 min |
| **Vardon 2008^45^** | Cohort | 449 | France | Women from 37 weeks in labour with NRFHR patterns | Low: FSp02 < 30%. Intermediate: 30-40% Normal: >40% | Low versus normal FSp02 | Yes | Electronic FHR monitoring (CTG) | UA ≤7.15, Diagnostic Accuracy of Fsp02 < 30% predicting poor neonatal status (composite variable: UA pH≤ 7.15, cord BD ≥12, 5min Apgar ≤7, NICU, secondary respiratory distress, death) Reduction in use of FBS since introduction of FPO | Range: Less than 50% to more than 70% |
| **Vitoratos 2002^46^** | Cohort | 85 | Greece | Primiparous women from 37 weeks in labour with NRFHR patterns | <30% for ≥10mins: EMCS | FSp02 <30% versus ≥ 30% | Yes | Electronic FHR monitoring (CTG) | UA≤7.15, NICU, CS rates and indications, Diagnostic accuracy of FSp02 < 30% predicting UA pH < 7.15 | 1st stage= 64.7 (SD=12.8%)  2nd stage=  65.4 (SD= 13.9%) |

Key: BE = base excess, CPR = cardiopulmonary resuscitation, CS = Cesarean section, CTG = cardiotocograph, ECG = electrocardiogram, EMCS = emergency Cesarean section, FBS = fetal blood sample, FPO = fetal pulse oximetry, FSpO2 = fetal oxygen saturation, IUGR = intrauterine growth restriction, IQR = interquartile range, NICU = neonatal intensive care unit, NRFHR = non-reassuring fetal heart rate, NRFS = non-reassuring fetal status, OD = operative delivery, OVD = operative vaginal delivery

RFHR = reassuring fetal heart rate, SCBU = special care baby unit, SD = Standard deviation, UA = umbilical artery, UV = umbilical vein

# Appendix 6. Correlation between fetal oxygen saturation and umbilical cord pH values

Table 1. Correlation between fetal oxygen saturation and umbilical artery pH

| **Study ID** | **Number of participants** | **Correlation coefficient** | **p-value** | **Note** |
| --- | --- | --- | --- | --- |
| **Kuhnert 2004^7^** | 73 | 0.84 | <0.001 | Correlation between FSp02 in last 10 minutes before birth and UA pH |
| **Carbonne 1997^14^** | 37 | 0.45 | 0.0051 | Correlation between FSp02 in last 10 minutes before birth and UA pH |
| **Chua 1999^15^** | 73 | No correlation (r value not reported) | No correlation (p-value not reported) | Correlation between mean FSp02 in last 10 minutes of labour and UA pH. No low FSp02 values or low UA pH values |
| **Fernandez Andres 2004^18^** | 80 | 0.573 | <0.01 | Correlation between FSp02 during active second stage of labour and scalp or UA pH |
| **Langer 1996^23^** | 40 | 0.55 | 0.0002 | Correlation between FSP02 in active second stage and UA pH |
| **Linhartova 2009^24^** | 150 | 0.54 | <0.0001 | Correlation between mean FSp02 and UA pH |
| **Markwitz 2000^47^** | 25 | 0.42 | 0.004 | Correlation between FSp02 in the last 30 minutes of the second stage of labour and UA pH |
| **McNamara 1992^28^** | 28 | 0.63 | 0.001 | Correlation before FSp02 and UV pH |
| **Salamalekis 1999^33^** | 48 | 0.76 | <0.05 | Correlation between FSp02 in second stage of labour and UA pH in cases with normal FHR patterns |
| **Salamalekis 1999^33^** | 20 | 0.78 | <0.05 | Correlation between FSp02 in second stage of labour and UA pH in cases with abnormal FHR patterns |
| **Tomialowicz 2007^43^** | 21 | No correlation (r-value not reported) | Non-significant (p-value not reported) | Correlation between mean FSP02 in the last 30 minutes of the second stage of labour and UV pH. No cases of FSp02 <30% |
| **Uchida 2015^44^** | 30 | 0.52 | 0.003 | Fetal tissue oximetry |

Table 2. Correlation between fetal oxygen saturation (FSP02) and venous umbilical cord (UV) pH

| **Study ID** | **Number of participants** | **Correlation coefficient** | **p-value** | **Note** |
| --- | --- | --- | --- | --- |
| **Carbonne 1997^14^** | 37 | 0.53 | 0.0007 | Correlation between FSp02 in last 10 minutes before birth and UA pH |
| **Chua 1999^15^** | 73 | No correlation (r value not reported) | No correlation (p-value not reported) | Correlation between mean FSp02 in last 10 minutes of labour and UV pH. No low FSP02 values or low UV pH values |
| **Langer 1996^23^** | 40 | 0.47 | 0.002 | Correlation between FSp02 in active second stage and UV pH |
| **Leszczynska-Gorzelak 2002^48^** | 28 | 0.38 | Non-significant (p-value not reported) | Correlation between FSp02 in the first stage of labour and UV pH. FSp02 >40% in all cases |
| **Leszczynska-Gorzelak 2002^48^** | 28 | -0.01 | Non-significant(p-value not reported) | Correlation between FSp02 in the second stage of labour and UV pH. FSp02 >40% in all cases |
| **Markwitz 2000^47^** | 24 | 0.49 | 0.006 | Correlation between FSp02 in the last 30 minutes of the second stage of labour and UV pH |
| **McNamara 1992^28^** | 28 | 0.57 | 0.002 | Correlation before FSp02 and UV pH |
| **Tomialowicz 2007^43^** | 21 | No correlation (r-value not reported) | Non-significant (p-value not reported) | Correlation between mean FSp02 in the last 30 minutes of the second stage of labour and UV pH. No cases of FSp02 < 30% |

**Key:** FSp02 = fetal oxygen saturation. UA = umbilical artery UA = umbilical vein

# Appendix 7: Additional Data

***The correlation between fetal oxygen saturation levels and umbilical artery or vein pH levels***

Seelbach-Gobel (2005)^36^ found that when FSp02 levels were above 60%, there was no significant change in umbilical artery pH levels (r=0.2). A slight decrease in umbilical artery pH was observed with FSp02 between 30-60% (r=0.13). A larger decrease in umbilical artery pH was noted when FSp02 fell below 30% (r=0.48), especially with prolonged exposure defined as more than 10 minutes. For episodes shorter than 10 minutes below 30% FSp02, no significant drop in pH below 7.1 was recorded. Conversely, in cases where FSp02 remained below 30% for over 10 minutes, more than half showed pH values below 7.2.

***The association between the addition of fetal oxygen saturation monitoring to fetal heart rate monitoring and umbilical artery lactate levels***

East (2006) found that umbilical artery lactate levels remained stable in neonates with normal FSp02 levels even when delivery was not expedited in response to non-reassuring fetal heart rate patterns (FSp02 + FHR monitoring: 216 cases, median UA lactate: 4.2mmol/L, No FSp02: 194 cases, median UA lactate: 4.2mmol/L, p = 0.605).^49^

***The addition of fetal oxygen saturation monitoring to fetal heart rate monitoring and cardiopulmonary resuscitation, hypoxic ischaemic encephalopathy and neonatal or intrapartum death***

East (2006) found no infants requiring CPR in either the group monitored with (n=305) or without (n=295) FSp02.^4^ Bloom (2006) reported no hypoxic ischaemic encephalopathy cases in the group with accessible FSp02 values (n=2,629) and one case in the group with blinded FSp02 values (n=2,712, p=1.00).^1^ Vardon (2008) reported a single neonatal death, occurring in the group with FSp02 levels between 30-40%, attributed to complications following a ventouse delivery.^45^ Nikolov (2004), Kuhnert (1998) and Csitari (2008) documented no instances of neonatal or intrapartum mortalities.^16, 21, 29^

***The association between fetal oxygen saturation less than 30% and umbilical artery lactate levels***

No study investigated the association between FSp02 less than 30% and our threshold of UA lactate > 4.8mmol/L. Luttkus (2003) noted a lower median FSp02 in infants with a lactate level of ≥ 5.5 mmol/l (n=13) compared those with a lactate less than 5.5 mmol/l (57% versus 70%, p<0.05).^27^ Hajek (2006), examined 114 high-risk mother-infant pairs and reported that an FSp02 threshold of less than 30% had a sensitivity of 100% and a specificity of 40% in predicting an UA lactate ≥3.7mmol/l.^20^ In comparison, CTG demonstrated a sensitivity of 100% and a lower specificity of 5%.

***The association between fetal oxygen saturation and umbilical cord oxygen saturation levels***

No study compared the association between FSp02 less than 30% and UA oxygen saturation less than 30% or umbilical vein oxygen saturation less than 55%. McNamara found a significant correlation between mean FSp02 during the second stage of labour and umbilical vein oxygen saturation (n=29 participants, r=0.59, p<0.001).^28^ However, no correlation was observed between FSp02 and UA oxygen saturation (r=0.63, p=0.001).

***The association between fetal oxygen saturation levels and fetal scalp pH levels***

Kuhnert (1998) found lower mean fetal scalp pH values in fetuses with FSp02 ≤ 30% (pH = 7.176 ± 0.017, n=13) compared to those with FSp02 > 30% (pH = 7.297 ± 0.058, p<0.0001, n=37)^21^. Kuhnert (2004) found a moderate correlation (r=0.488, p=0.005), while Butterwegge (1997) observed a stronger correlation (r = 0.62, p < 0.001, n = 34).^7, 50^ Fernandez Andres (2004) reported correlations during labour (r = 0.382, p < 0.01) and particularly in the second stage of labour (r = 0.573, p < 0.01).^18^ Four studies reported a significant correlation between FSp02 and scalp pH, with respective results of Kuhnert (2004): r = 0.488, p = 0.005; Butterwegge (1997): r = 0.62, p < 0.001, n = 34; Fernandez Andres (2004): entire labour = (r = 0.382, p < 0.01) second stage of labour (r = 0.573, p < 0.01) and Langer (1996): r = 0.52, p < 0.05, n = 21. However, Rijnders (2002) observed no significant correlation between FSp02 and fetal scalp or UA pH (r = -0.02, p = 0.9).

Kuhnert (2001) highlighted a negative correlation between FSp02 ≤ 30% and pH changes, observing a pH drop of 0.02 units in 10 minutes at FSp02 ≤ 30%, compared to a drop of 0.002 units at FSp02 between 31-60%.^22^ A more pronounced pH drop (> 0.05 units) was noted when FSp02 remained ≤ 30% for over 10 minutes.

Seelbach-Gobel (1999) found that FSp02 ≤ 30% for 10 minutes or more predicted a decrease in scalp pH of more than 0.05 with high sensitivity (100%) and specificity (65%).^35^ Further, Seelbach-Gobel (2005) reported that in cases where scalp pH was less than 7.20, 70% had FSp02 ≤ 30% for at least 10 minutes, and when scalp pH was below 7.15, all cases had FSp02 ≤ 30% for the same duration.^36^

Luttkus (1996) and Luttkus (2002) reported a strong positive correlation between FSp02 and oxygen saturation from haemoximetry from the fetal scalp in cases with an umbilical artery pH ≤ 7.16 and base excess ≤ -9.4mmol/l ([r=0.74, total number and p-value not reported] and [n=18, r=0.72, p=0.002] respectively).^25, 26^ Notably, no studies have reported on the association between FSp02 and scalp lactate.

***The addition of fetal oxygen saturation monitoring to fetal heart rate monitoring and operative delivery rates***

There was no difference in the rates of operative delivery, encompassing both instrumental vaginal and Cesarean births, between the two cohorts (5 RCTs, 3,605 cases with FSp02 + FHR, 3,672 cases without FSp02, OR = 0.96, 95% CI = [0.69–1.33], I² = 81%, p = 0.80), see Figure 12).^1, 4, 5, 7, 8, 19^

***Methods used to measure fetal oxygen saturation in included studies***

While the majority of studies characterised low FSp02 as being <30%, the specified duration for which the FSp02 must remain under this threshold was not consistent. Forty studies (85.1%) used the Nellcor pulse oximeter, which was positioned between the fetal cheek or scalp and the uterine wall.^14^ Three studies (6.4%) used the Corometrics oxicardiotocography series 120 with a Nellcor FS-14B fetal oxygen sensor.^37, 38, 46^ One study used the OBS-500 fetal pulse oximetry system, placing it between the fetal scapulae and the uterine wall.^27^ Uchida (2016) used the Uchida probe to measure fetal tissue oximetry and positioned the probe on the fetal scalp.^51^ Stiller (2002) also placed the probe on the fetal scalp using the Zurich Reflectance Pulse Oximetry System.^42^ Tables one and two in Appendix 5 demonstrate variability in pulse oximetry signal quality across studies. Overall mean signal quality was 74.9% (standard deviation [SD] = 14.7%) for the monitoring period. During the first stage of labour, mean FSp02 was 73.41% (SD = 11.02), which decreased to 61.91% (SD = 18.67) in the second stage (mean difference = 11.50%, 95% CI = [1.55, 21.44], t = 2.829, p = 0.030). A strong correlation was observed between the first and second stage of labour (r = 0.861, p = 0.013).


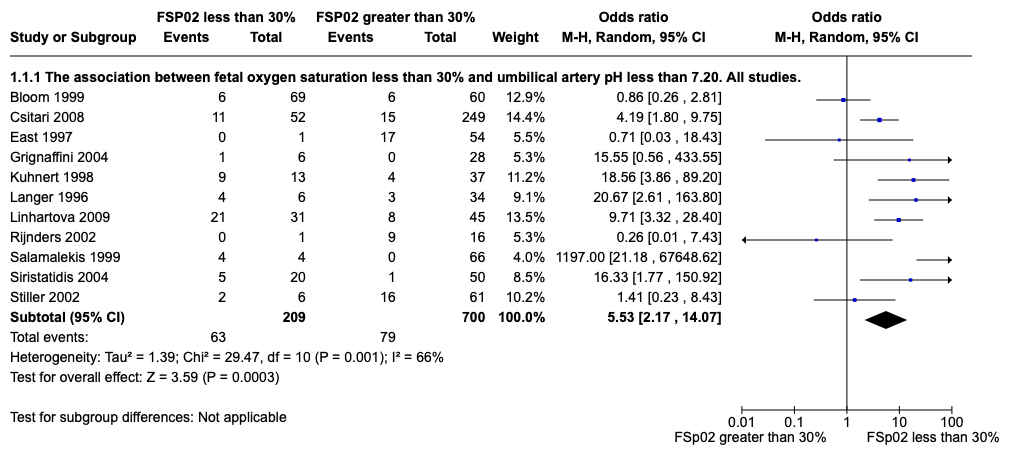


Figure 1. The association between fetal oxygen saturation less than 30% and umbilical artery pH less than 7.20. All studies

**
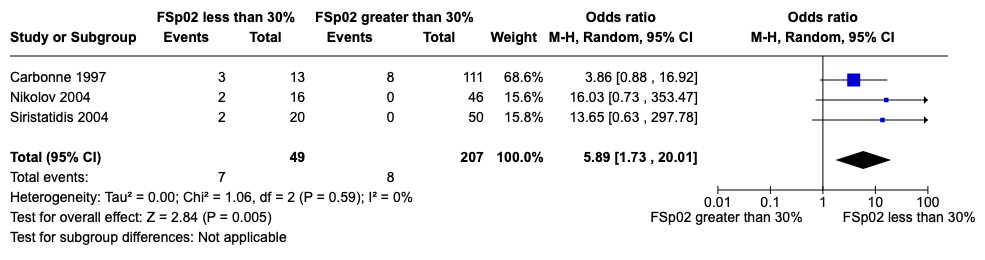
**

Figure 2. The association between fetal oxygen saturation less than 30% and admissions to the neonatal intensive care unit


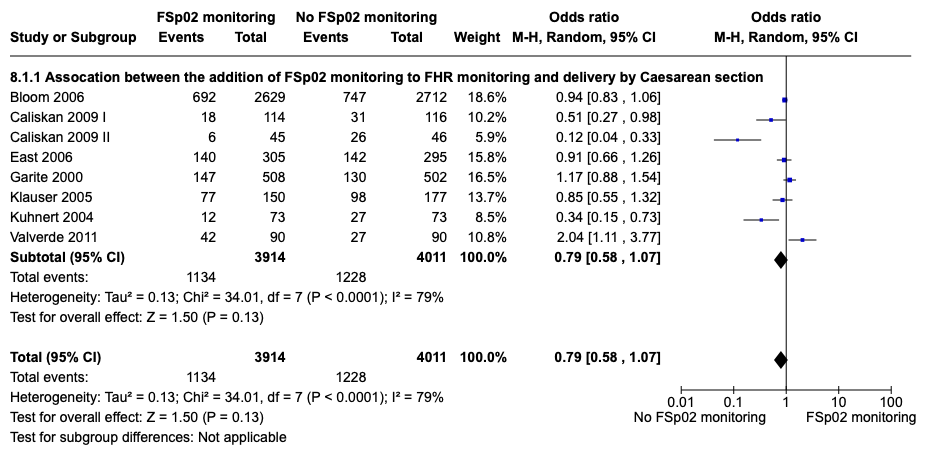


Figure 3. The association between addition of fetal oxygen saturation monitoring to fetal heart rate monitoring and delivery by Cesarean section

**
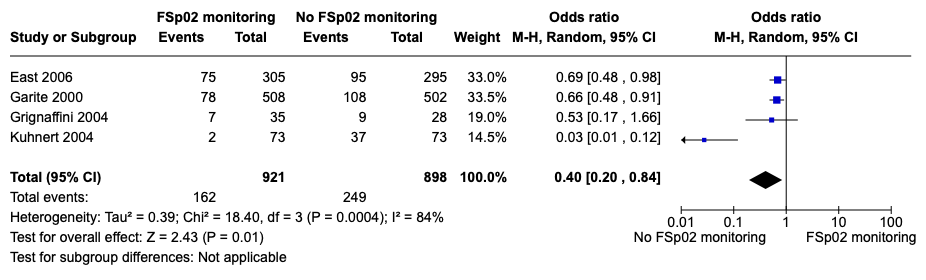
**

Figure 4. The association between addition of fetal oxygen saturation monitoring to fetal heart rate monitoring and operative vaginal deliveries for non-reassuring fetal status


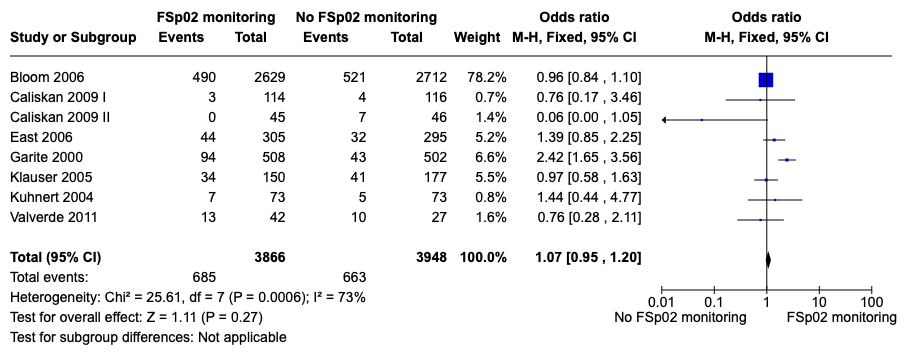


Figure 5. The association between addition of fetal oxygen saturation monitoring to fetal heart rate monitoring and Cesarean section for dystocia in labour

**
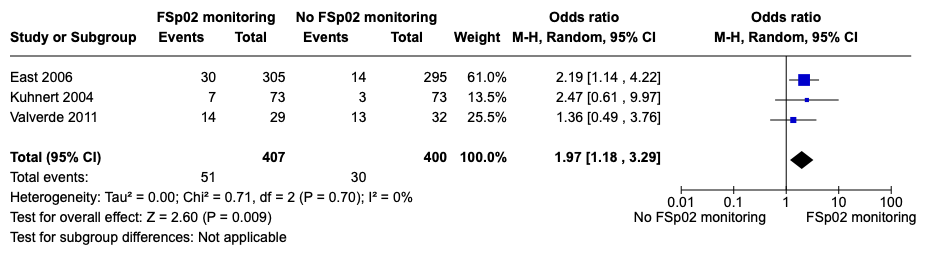
**

Figure 6. The association between addition of fetal oxygen saturation monitoring to fetal heart rate monitoring and operative vaginal deliveries for dystocia in labour

**
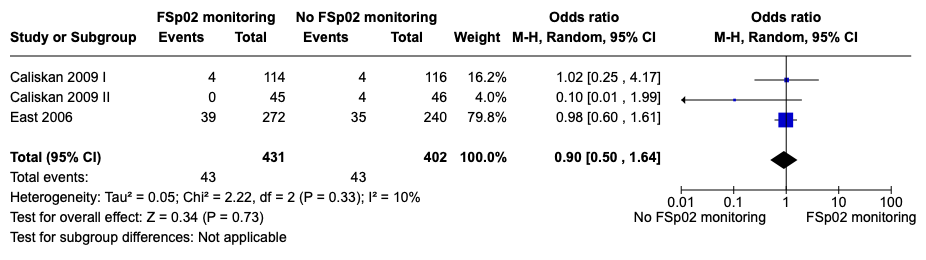
**

Figure 7. The association between addition of fetal oxygen saturation monitoring to fetal heart rate monitoring and umbilical artery pH less than 7.15

**
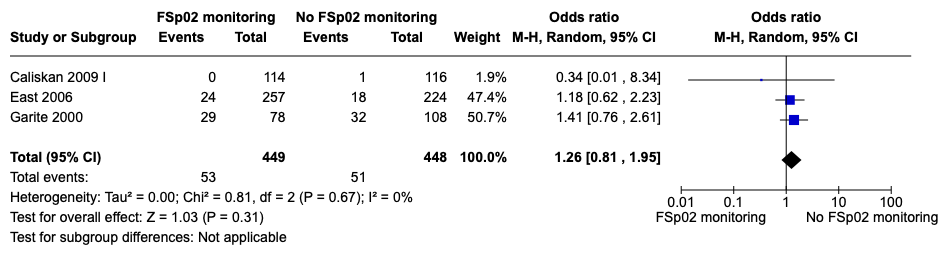
**

Figure 8. The association between addition of fetal oxygen saturation monitoring to fetal heart rate monitoring and umbilical artery base excess of less than or equal to -10mmol/L

**
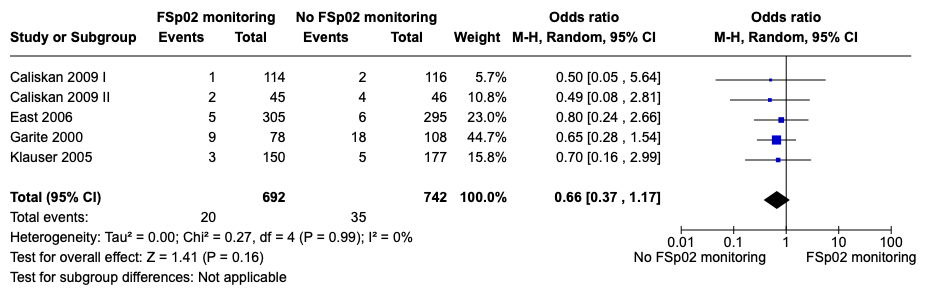
**

Figure 9. The association between addition of fetal oxygen saturation monitoring to fetal heart rate monitoring and 5 minute Apgar scores of less than 7

**
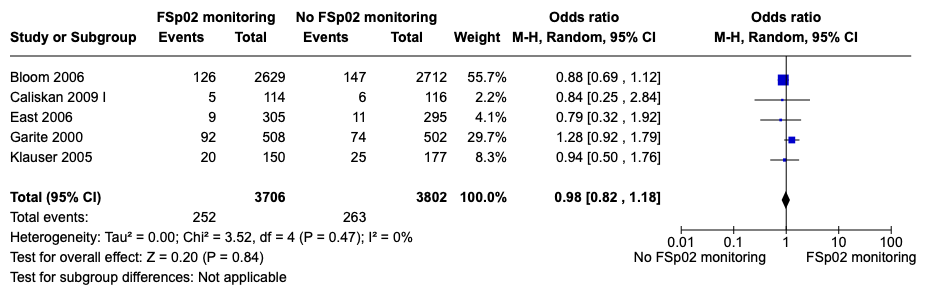
**

Figure 10. The association between addition of fetal oxygen saturation monitoring to fetal heart rate monitoring and admissions to the neonatal intensive care unit

**
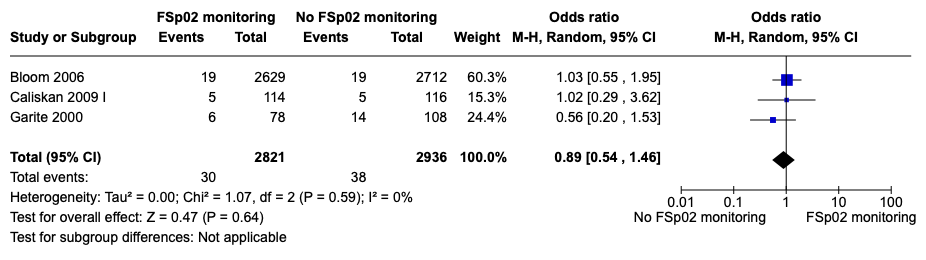
**

Figure 11. The association between addition of fetal oxygen saturation monitoring to fetal heart rate monitoring and neonatal intubation

**
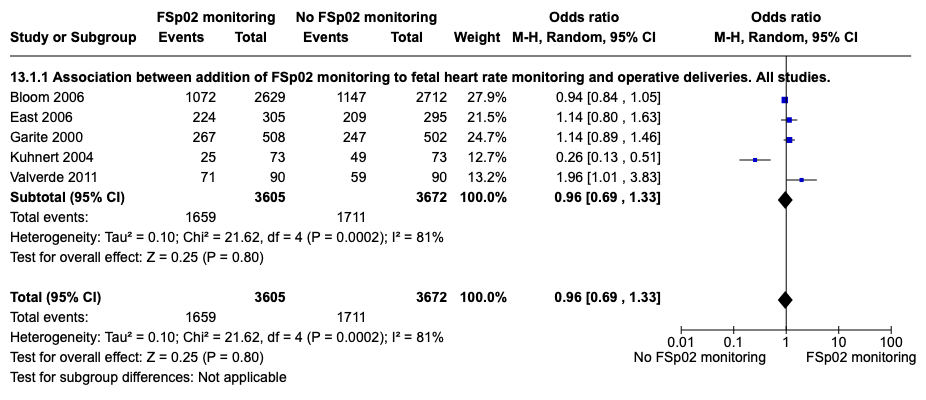
**

Figure 12. The association between addition of fetal oxygen saturation monitoring to fetal heart rate monitoring and operative deliveries (Cesarean sections and operative vaginal deliveries)

# Appendix 8. Quality Assessment and GRADE Analysis

We assessed study quality using the RoB tool with the following scale:

- 'Good quality': All criteria are met, indicating a low risk of bias.
- 'Fair quality': The study has one domain with a high risk of bias or two unclear risks, but these do not substantially threaten the validity of the outcome.
- 'Poor quality': The study has one domain with a high risk of bias or two unclear risks that likely affect the outcome, or it has significant limitations that could invalidate the results.
- Additionally, studies with two or more high or unclear risks are also rated as poor quality.

Studies were assessed according to the NOS as “good quality” if they scored between 7 and 9, “fair quality” if they scored between 5 and 6 and “poor quality” if they scored between 0 and 4.^52^


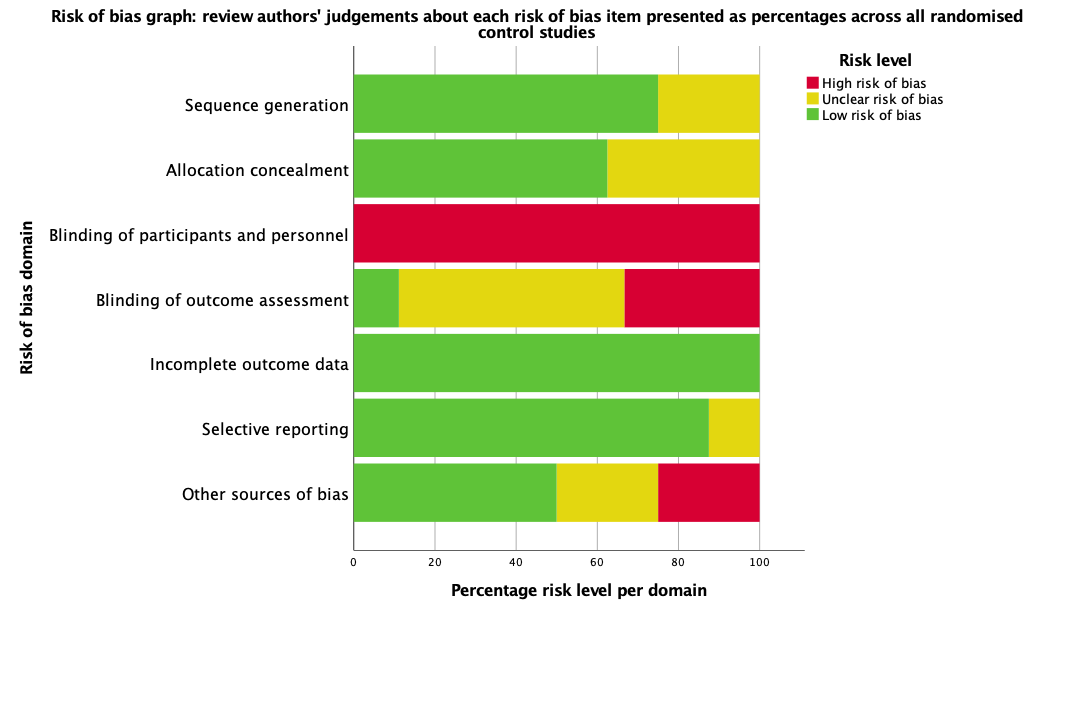


Figure 1. Methodological quality of randomized control studies in systematic review of association between fetal pulse oximetry and adverse perinatal and long-term outcomes


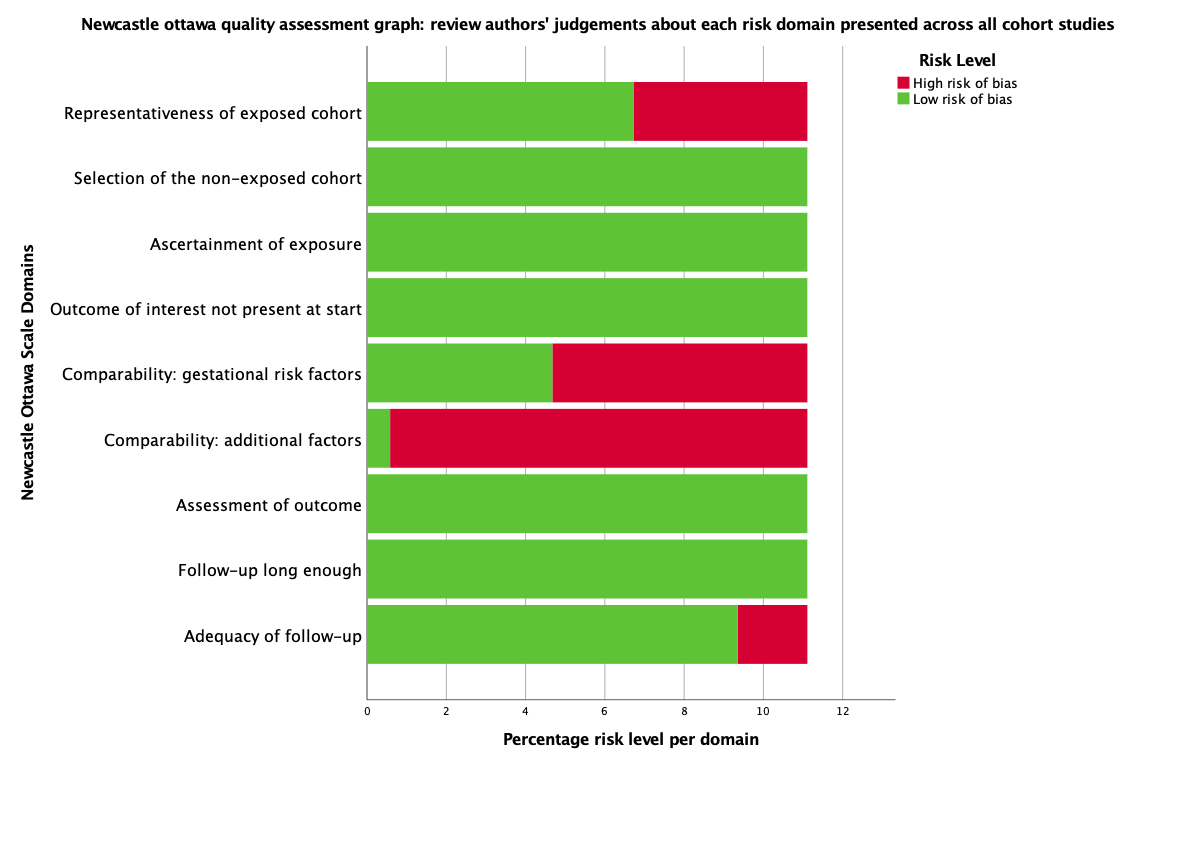


Figure 2. Methodological quality of non-randomized studies in systematic review of association between fetal pulse oximetry and adverse perinatal and long-term outcome

Table 1. Risk of bias summary: review authors’ judgements about each risk of bias item for each included randomized control study

|  | Sequence generation | Allocation concealment | Blinding of participants and personnel | Blinding of outcome assessment | Incomplete outcome data | Selective reporting | Other bias |
| --- | --- | --- | --- | --- | --- | --- | --- |
| Bloom 2006^1^ |  |  |  |  |  |  |  |
| Caliskan 2009 I^2^ |  |  |  |  |  |  |  |
| Caliskan 2009 II^3^ |  |  |  |  |  |  |  |
| East 2006^4^ |  |  |  |  |  |  |  |
| Garite 2000^5^ |  |  |  |  |  |  |  |
| Klauser 2005^6^ |  |  |  |  |  |  |  |
| Kuhnert 2004^53^ |  |  |  |  |  |  |  |
| Valverde 2010^54^ |  |  |  |  |  |  |  |

Key: Low risk of bias =. Unclear risk of bias = High risk of bias =

Table 2. Risk of bias summary: review authors’ judgements about each risk of bias item

| Newcastle-Ottawa Scale Quality Assessment Scale for Cohort Studies | | | | | | | | | | | | | | | | | | | | | | | |
| --- | --- | --- | --- | --- | --- | --- | --- | --- | --- | --- | --- | --- | --- | --- | --- | --- | --- | --- | --- | --- | --- | --- | --- |
|  | | **Selection** | | | | | | **Comparability** | | | | | **Outcome** | | | | | | | | | | |
|  | **Representativeness of the exposed cohort** | | | **Selection of the nonexposed cohort** | **Ascertainment of exposure** | | **Outcome of interest not present at start of study** | | **Comparability of cohorts** | | | | **Assessment of outcome** | **Sufficient follow-up time** | | | **Adequacy of follow-up** | | | **Total**  **out of 9** | | | |
| Study ID |  |  |  |  |  |  |  |  | **Antenatal or pregnancy risk factors** | | **Additional factors** | |  |  |  |  |  |  |  |  |  |  |  |
| Bakr 2005^9^ | | 0 | | * | * | | * | | * | | 0 | | * | * | | | * | | | | | 7 | |
| Bloom 1999^11^ | | * | | * | * | | * | | 0 | | 0 | | * | * | | | * | | | | | 7 | |
| Butterwegge 1997^12^ | | * | | * | * | | * | | * | | 0 | | * | * | | | 0 | | | | | 7 | |
| Carbonne 1994^13^ | | * | | * | * | | * | | * | | 0 | | * | * | | | * | | | | | 8 | |
| Carbonne 1997^14^ | | * | | * | * | | * | | * | | 0 | | * | * | | | * | | | | | 8 | |
| Chua 1999^15^ | | 0 | | * | * | | * | | 0 | | 0 | | * | * | | | * | | | | | 6 | |
| Csitari 2008^16^ | | * | | * | * | | * | | 0 | | 0 | | * | * | | | * | | | | | 7 | |
| East 1997^17^ | | * | | * | * | | * | | 0 | | 0 | | * | * | | | 0 | | | | | 6 | |
| Fernandez Andres 2004^18^ | | * | | * | * | | * | | * | | 0 | | * | * | | | * | | | | | 8 | |
| Grignaffini 2004^19^ | | * | | * | * | | * | | * | | 0 | | * | * | | | * | | | | | 8 | |
| Hajek 2006^20^ | | 0 | | * | * | | * | | 0 | | 0 | | * | * | | | * | | | | | 6 | |
| Kuhnert 1998^21^ | | 0 | | * | * | | * | | 0 | | 0 | | * | * | | | * | | | | | 6 | |
| Kuhnert 2001^22^ | | 0 | | * | * | | * | | 0 | | 0 | | * | * | | | 0 | | | | | 5 | |
| Langer 1996^23^ | | * | | * | * | | * | | 0 | | * | | * | * | | | * | | | | | 8 | |
| Leszczynska-Gorzelak 2002^48^ | | 0 | | * | * | | * | | 0 | | 0 | | * | * | | | 0 | | | | | 5 | |
| Linhartova 2009^24^ | | * | | * | * | | * | | 0 | | 0 | | * | * | | | * | | | | | 7 | |
| Luttkus 1996^25^ | | 0 | | * | * | | * | | 0 | | 0 | | * | * | | | 0 | | | | | 5 | |
| Luttkus 2002^26^ | | 0 | | * | * | | * | | 0 | | 0 | | * | * | | | * | | | | | 6 | |
| Luttkus 2003^27^ | | * | | * | * | | * | | * | | 0 | | * | * | | | * | | | | | 8 | |
| Markwitz 2000^47^ | | 0 | | * | * | | * | | * | | 0 | | * | * | | | * | | | | | 7 | |
| McNamara 1992^28^ | | 0 | | * | * | | * | | 0 | | 0 | | * | * | | | * | | | | | 6 | |
| Nikolov 2004^29^ | | * | | * | * | | * | | 0 | | 0 | | * | * | | | * | | | | | 7 | |
| Nonnenmacher 2010^30^ | | 0 | | * | * | | * | | 0 | | 0 | | * | * | | | * | | | | | 6 | |
| Rijnders 2002^31^ | | 0 | | * | * | | * | | 0 | | 0 | | * | * | | | * | | | | | 6 | |
| Roztocil 2000^32^ | | * | | * | * | | * | | 0 | | 0 | | * | * | | | * | | | | | 7 | |
| Salamalekis 1999^33^ | | * | | * | * | | * | | * | | 0 | | * | * | | | * | | | | | 8 | |
| Seelbach-Gobel 1994^34^ | | 0 | | * | * | | * | | 0 | | 0 | | * | * | | | 0 | | | | | 5 | |
| Seelbach-Gobel 1999^35^ | | * | | * | * | | * | | * | | 0 | | * | * | | | * | | | | | 8 | |
| Seelbach-Gobel 2005^36^ | | 0 | | * | * | | * | | 0 | | 0 | | * | * | | | * | | | | | 6 | |
| Siristatidis 2003^37^ | | * | | * | * | | * | | * | | 0 | | * | * | | | * | | | | | 8 | |
| Siristatidis 2004^38^ | | * | | * | * | | * | | * | | * | | * | * | | | * | | | | | 9 | |
| Skoczylas 2003^39^ | | * | | * | * | | * | | * | | 0 | | * | * | | | * | | | | | 8 | |
| Sobotkova 2004^40^ | | * | | * | * | | * | | * | | 0 | | * | * | | | * | | | | | 8 | |
| Stiller 2002^42^ | | * | | * | * | | * | | * | | 0 | | * | * | | | * | | | | | 8 | |
| Tomialowicz 2007^43^ | | * | | * | * | | * | | 0 | | 0 | | * | * | | | * | | | | | 7 | |
| Uchida 2015^44^ | | 0 | | * | * | | * | | * | | 0 | | * | * | | | * | | | | | 7 | |
| Vardon 2008^45^ | | * | | * | * | | * | | 0 | | 0 | | * | * | | | * | | | | | 7 | |
| Vitoratos 2002^46^ | | * | | * | * | | * | | 0 | | 0 | | * | * | | | * | | | | | 7 | |
| Newcastle Ottawa Quality Assessment Scale for Case-Control Studies | | | | | | | | | | | | | | | | | | | | |  | | |
|  | | | **Adequate case definition** | **Representativeness of cases** | | **Selection of controls** | | **Definition of controls** | | **Comparability** | | **Ascertainment of exposure** | | **Method of ascertainment** | | **Non-response rate** | | | **Total out of 9** | | | |  |
| Biringer 2011^10^ | | | ***** | **0** | | ***** | | ***** | | ****** | | ***** | | ***** | | **0** | |  | | | 7 | | |
|  | | |  |  | |  | |  | |  | |  | |  |  | | |  | | |  | | |

Table 3. GRADE Analysis: Association between FSp02 less than 30% and Adverse Perinatal Outcomes compared to FSp02 greater than 30%

| **Certainty assessment** | | | | | | | **№ of patients** | | **Effect** | | **Certainty** | **Importance** |
| --- | --- | --- | --- | --- | --- | --- | --- | --- | --- | --- | --- | --- |
| **№ of studies** | **Study design** | **Risk of bias** | **Inconsistency** | **Indirectness** | **Imprecision** | **Other considerations** | **Association between FSp02 <30% and Adverse Perinatal Outcomes** | **[placebo]** | **Relative (95% CI)** | **Absolute (95% CI)** |  |  |
| **The Association between FSp02 less than 30% and umbilical artery pH less than 7.15** | | | | | | | | | | | | |
| 9 | non-randomized studies | serious^a^ | serious^b^ | not serious | not serious | very strong association | 111/229 (48.5%) | 121/875 (13.8%) | OR 7.86 (3.29 to 18.75) | 420 more per 1,000 (from 207 more to 612 more) | ⨁⨁◯◯ Low | IMPORTANT |
| **The association between FSp02 less than 30% and umbilical artery pH less than 7.20** | | | | | | | | | | | | |
| 11 | non-randomized studies | serious^d^ | serious^b^ | not serious | not serious | very strong association | 63/209 (30.1%) | 79/700 (11.3%) | OR 5.53 (2.17 to 14.07) | 300 more per 1,000 (from 103 more to 529 more) | ⨁⨁◯◯ Low | IMPORTANT |
| **The association between FSp02 less than 30% and 5 minute Apgar score less than 7** | | | | | | | | | | | | |
| 6 | non-randomized studies | not serious | not serious | not serious | serious^c^ | very strong association | 25/104 (24.0%) | 10/585 (1.7%) | **OR 16.63** (5.64 to 49.01) | **207 more per 1,000** (from 72 more to 443 more) | ⨁⨁⨁◯ Moderate | IMPORTANT |
| **The association between FSP02 less than 30% and NICU admission** | | | | | | | | | | | | |
| 3 | non-randomized studies | not serious | not serious | not serious | serious^c^ | very strong association | 7/49 (14.3%) | 8/207 (3.9%) | OR 5.89 (1.73 to 20.01) | 153 more per 1,000 (from 26 more to 407 more) | ⨁⨁⨁◯ Moderate | IMPORTANT |

**CI:** confidence interval; **OR:** odds ratio

#### Explanations

a. 5 out of 9 studies were "fair" quality as assessed by the Newcastle Ottawa Scale

b. Variance of point estimates across studies with large I2 value and low p-value

c. Included studies contained wide 95% confidence intervals

d. 4 out of 11 studies were "fair" quality as assessed by the Newcastle Ottawa Scale

Table 4. GRADE Analysis: The association between the addition of FSp02 monitoring to standard monitoring and adverse perinatal outcomes compared to standard monitoring.

| **Certainty assessment** | | | | | | | **№ of patients** | | **Effect** | | **Certainty** | **Importance** |
| --- | --- | --- | --- | --- | --- | --- | --- | --- | --- | --- | --- | --- |
| **№ of studies** | **Study design** | **Risk of bias** | **Inconsistency** | **Indirectness** | **Imprecision** | **Other considerations** | **The association between the addition of FSp02 monitoring to standard monitoring and adverse perinatal outcomes** | **[placebo]** | **Relative (95% CI)** | **Absolute (95% CI)** |  |  |
| **The association between the addition of FSp02 monitoring to standard monitoring and umbilical artery pH < 7.0** | | | | | | | | | | | | |
| 4 | randomized trials | serious | serious^a^ | not serious | very serious^b^ | none | 18/3559 (0.5%) | 22/3631 (0.6%) | OR 0.91 (0.48 to 1.73) | 1 fewer per 1,000 (from 3 fewer to 4 more) | ⨁◯◯◯ Very low | IMPORTANT |
| **The association between the addition of FSp02 monitoring to standard monitoring and umbilical artery pH < 7.15** | | | | | | | | | | | | |
| 3 | randomized trials | serious | serious^a^ | not serious | very serious^b^ | none | 43/431 (10.0%) | 43/402 (10.7%) | OR 0.90 (0.50 to 1.64) | 10 fewer per 1,000 (from 50 fewer to 57 more) | ⨁◯◯◯ Very low | IMPORTANT |
| **The association between the addition of FSp02 monitoring to standard monitoring and umbilical artery base excess ≤ -10mmol/l** | | | | | | | | | | | | |
| 3 | randomized trials | serious | not serious | not serious | very serious^b^ | none | 53/449 (11.8%) | 51/448 (11.4%) | OR 1.26 (0.81 to 1.95) | 25 more per 1,000 (from 20 fewer to 86 more) | ⨁◯◯◯ Very low | IMPORTANT |
| **The association between the addition of FSp02 monitoring to standard monitoring and 5 minute Apgar score less than 7** | | | | | | | | | | | | |
| 5 | randomised trials | serious | not serious | not serious | very serious^b^ | none | 20/692 (2.9%) | 35/742 (4.7%) | OR 0.66 (0.37 to 1.17) | 16 fewer per 1,000 (from 29 fewer to 8 more) | ⨁◯◯◯ Very low | IMPORTANT |
| **The association between the addition of FSp02 monitoring to standard monitoring and NICU admission** | | | | | | | | | | | | |
| 5 | randomized trials | serious | not serious | not serious | very serious^b^ | none | 252/3706 (6.8%) | 263/3802 (6.9%) | OR 0.98 (0.82 to 1.18) | 1 fewer per 1,000 (from 12 fewer to 11 more) | ⨁◯◯◯ Very low | IMPORTANT |
| **The association between the addition of FSp02 monitoring to standard monitoring and neonatal intubation** | | | | | | | | | | | | |
| 3 | randomized trials | serious | not serious | not serious | very serious^b^ | none | 30/2821 (1.1%) | 38/2936 (1.3%) | OR 0.89 (0.54 to 1.46) | 1 fewer per 1,000 (from 6 fewer to 6 more) | ⨁◯◯◯ Very low | IMPORTANT |
| **The association between the addition of FSp02 monitoring to standard monitoring and Cesarean section** | | | | | | | | | | | | |
| 8 | randomized trials | serious | serious^a^ | not serious | very serious^b^ | none | 1134/3914 (29.0%) | 1228/4011 (30.6%) | OR 0.79 (0.58 to 1.07) | 48 fewer per 1,000 (from 102 fewer to 15 more) | ⨁◯◯◯ Very low | IMPORTANT |
| **The association between the addition of FSp02 monitoring to standard monitoring and Cesarean section for non-reassuring fetal status** | | | | | | | | | | | | |
| 8 | randomized trials | serious | serious^a^ | not serious | very serious^b^ | none | 341/3866 (8.8%) | 457/3948 (11.6%) | OR 0.59 (0.40 to 0.86) | 44 fewer per 1,000 (from 66 fewer to 15 fewer) | ⨁◯◯◯ Very low | IMPORTANT |

**CI:** confidence interval; **OR:** odds ratio

#### Explanations

a. Wide variance of point estimates across studies

b. The confidence intervals cross the clinical decision threshold.

# Appendix 9. Analyses

Subgroup analyses for the association of fetal oxygen saturation less than 30% and adverse perinatal outcomes

A subgroup analysis was performed when examining the association between fetal oxygen saturation less than 30% and UA pH less than 7.15 where Biringer 2011 was excluded as it is a case-control study to include cohort studies only.^10^ The results were unchanged with FSp02 less than 30% being associated with an UA pH less than 7.15 (Eight cohort studies, 207 cases of FSp02 < 30%, 830 cases of FSp02 > 30%, OR = 8.18, 95% CI = [2.99, 22.34], p = 0.0004, I^2^ = 74%, see Figure 1) .

The inclusion of “good quality” studies only for the analyses examining the association between FSp02 less than 30% and UA pH less than 7.20 and pH less than 7.15 did not change results: (1) UA < 7.20: (OR = 8.17, 95% CI = [2.07, 32.36], p = 0.003, I^2^ = 73%) (2) UA pH < 7.15: (OR = 6.59, 95% CI = [2.65, 16.39, p < 0.0001, I^2^ = 74%), see Figures 2 and 3.

We performed a subgroup analysis where we included “good quality” studies only investigating the association between FSp02 less than 30% and 5 minute Apgar score less than 7 and the results were unchanged (OR = 24.45, 95% CI = [9.28, 64.41], p<0.00001, I^2^ = 0%, see Figure 7).

Subgroup analyses on the addition of FSp02 monitoring to FHR monitoring and adverse perinatal and delivery outcomes

As the analysis comparing the addition of FSp02 monitoring to FHR monitoring to FHR without FSp02 for detecting UA pH less than 7.20 included an RCT and two non-randomised studies we conducted a subgroup analysis omitting the RCT, see Figure 6. The results were still non-significant (2 studies, 115 cases with FSp02 + FHR, 108 cases with FHR monitoring without FPO, OR = 1.04, 95% CI = [0.27, 4.00], p = 0.95, I^2^ = 64%).

A subgroup analysis was performed excluding Bloom 2006 from the analysis assessing the impact of adding FSp02 measurement to FHR measurement in terms of Cesarean section rates due to its allowance for individual clinical management decisions rather than standardizing delivery expedience based on FSp02 values, and also omitting Valverde 2011 for comparing FPO with FHR and fetal ECG monitoring, rather than the specified FPO with FHR monitoring against FHR monitoring alone with or without the use of FBS (six RCTs with 1,195 cases with FSp02 monitoring and 1,209 without FSp02 monitoring, OR = 0.61, 95% = CI [0.39,0.96], I^2^ = 81%, p = 0.03), see Figure 8.

We conducted a subgroup analysis evaluating the effect of FSp02 monitoring combined with FHR monitoring on the rates of operative delivery, excluding studies that did not standardise delivery decisions based on FSp02 values (Bloom 2006) and those comparing FPO with FHR and fetal ECG monitoring (Valverde 2011), encompassing both instrumental vaginal and Cesarean births. No significant effect was noted on operative delivery rates (OR= 0.76, 95% CI = [0.39–1.45], I² = 88%, p= 0.40), see Figure 9.

We excluded Bloom 2006 and Valverde 2011 when examining the association between the addition of FSp02 monitoring to FHR monitoring and CS for NRFS, due to the methodological considerations outlined above, further strengthened the evidence for the beneficial effect of FPO, showing a more pronounced reduction in the odds ratio (OR = 0.47, 95% CI = [0.30–0.75]) with a significant overall effect (z = 3.19, p = 0.001) and a moderate level of heterogeneity (I² = 64%) (See Figure 10).^1, 55^

In terms of performing subgroup analyses for gestational age, the majority of included studies defined “term” as either greater than 36 or 37 weeks. We performed subgroup analyses where we excluded studies that included infants less than 36 weeks (n=4). The results were unchanged. See figures 13-17.

In a subgroup analysis analysing the association between umbilical artery pH < 7.20 and FSp02 < 30% for 10 minutes compared to FSp02 > 30% or < 30% for less than 10 mins, a low FSp02 for 10 minutes was associated with an UA pH < 7.20 (Three cohort studies, 96 cases of low FSp02%, 331 cases of normal FSp02, OR = 7.63, 95% CI = [3.35, 17.38], p < 0.001, I^2^ = 39%, see Appendix G. Figure 11, p.107). Similarly, in a subgroup analysis analysing the association between umbilical artery pH < 7.15 and FSp02 < 30% for 10 minutes compared to FSp02 > 30% or < 30% for less than 10 mins, a low FSp02 for 10 minutes was associated with an UA pH < 7.15 (Four cohort studies, 72 cases of low FSp02%, 228 cases of normal FSp02, OR =18.54, 95% CI = [5.63, 60.99], p < 0.001, I^2^ = 46%, see Appendix G. Figure 12. Notably, in both analyses investigating the association between low UA pH and FSp02 < 30% when the duration of low FSp02 is defined as 10 minutes or greater, there is a higher odds ratio and lower heterogeneity than when shorter durations are also included.

We performed subgroup analyses where we excluded studies that defined abnormal FSp02 as ≤30%. We repeated the analyses (i) association between fetal oxygen saturation less than 30% and UA pH <7.15 (Figure 18) (ii) association between fetal oxygen saturation less than 30% and UA pH <7 (Figure 19) (iii) association between fetal oxygen saturation less than 30% and 5 minute Apgar score < 7 (Figure 20) (iv) association between fetal oxygen saturation less than 30% and admission to NICU (Figure 21).


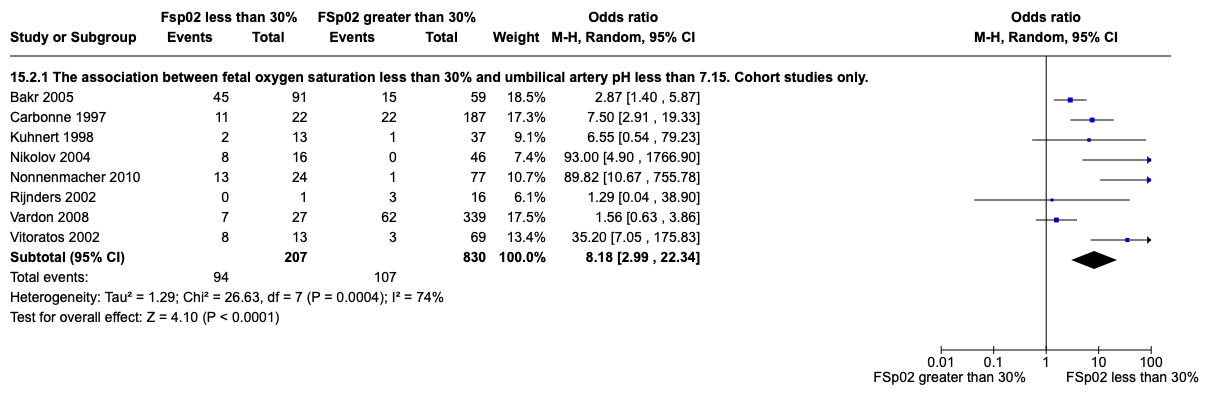


Figure 1. The association between fetal oxygen saturation less than 30% and umbilical artery pH less than 7.15. Cohort studies only.

**
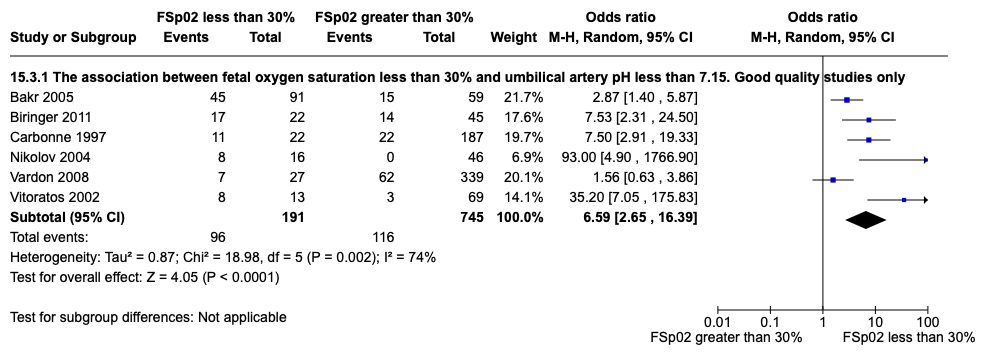
**

Figure 2. The association between fetal oxygen saturation less than 30% and umbilical artery pH less than 7.15. Good quality studies only.


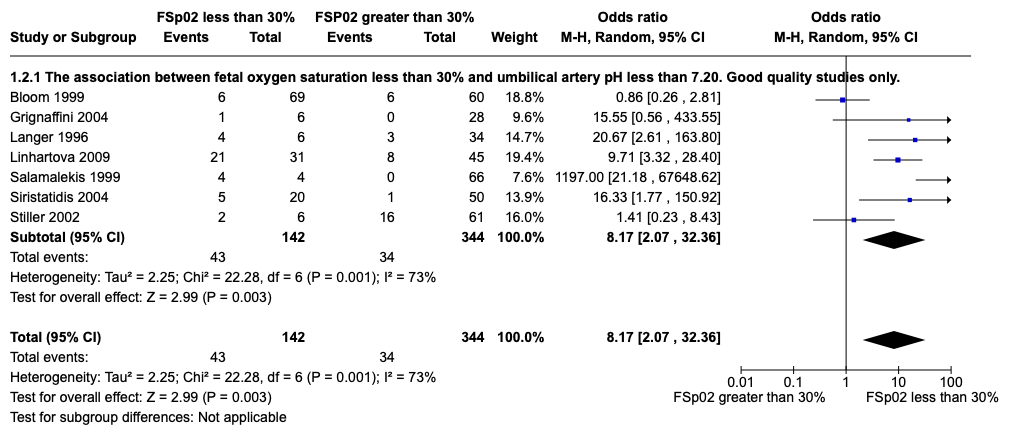


Figure 3. The association between fetal oxygen saturation less than 30% and umbilical artery pH less than 7.20. Good quality studies only.

***Assessment for Publication Bias***

As more than ten studies were included in the analyses examining the association between FSp02 less than 30% and UA pH less than 7.20 and pH less than 7.15, funnel plots and Egger’s test were utilised to evaluate the presence of publication bias, see Figures 4 and 5. The symmetry observed in these funnel plots suggests that publication bias is not a significant concern. Egger's test did not indicate the presence of publication bias in the analysis examining the association between low FSp02 levels and UA pH less than 7.15 (intercept = 0.391, standard error = 0.6864, t = 0.570, p = 0.586, 95% CI = [-1.232-2.015]). Similarly, Egger's test to assess the analysis examining the association between low FSp02 levels and UA pH < 7.20 continued to show no significant evidence of publication bias (intercept = 0.967, standard error = 1.1774, t = 0.821, p = 0.433), with the 95% confidence interval for the intercept spanning from -1.697 to 3.630.

**
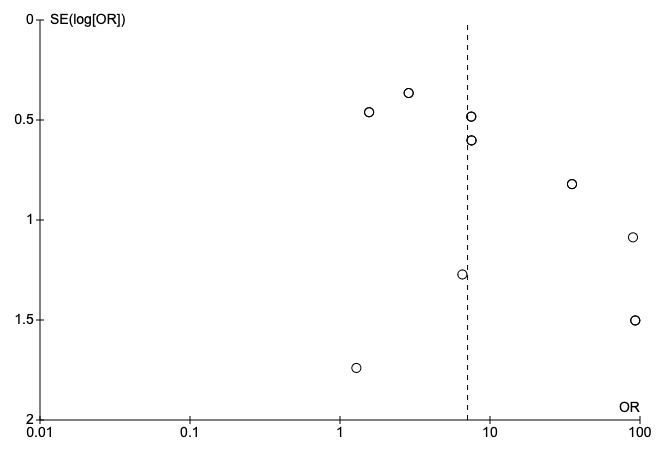
**

Figure 4. Funnel Plot: The association between fetal oxygen saturation less than 30% and umbilical artery pH less than 7.15.

**
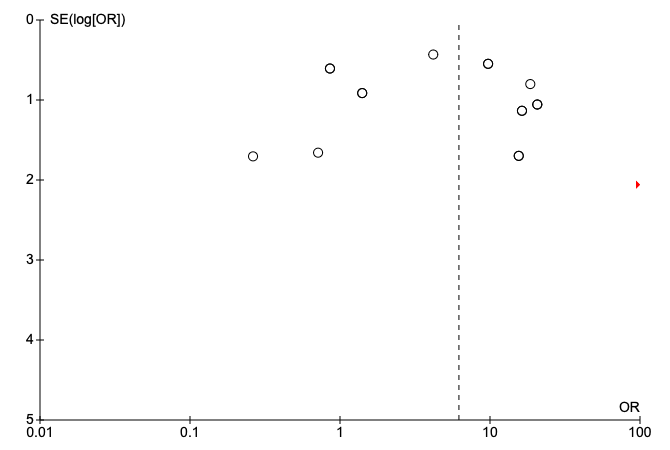
**

Figure 5. Funnel Plot: The association between fetal oxygen saturation less than 30% and umbilical artery pH less than 7.20


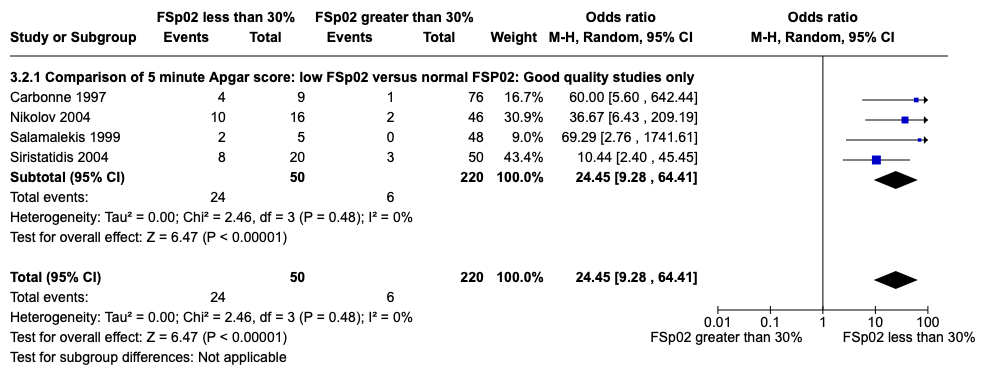


Figure 7. The association between fetal oxygen saturation less than 30% and 5 minute Apgar score less than 7. Good quality studies only.

**
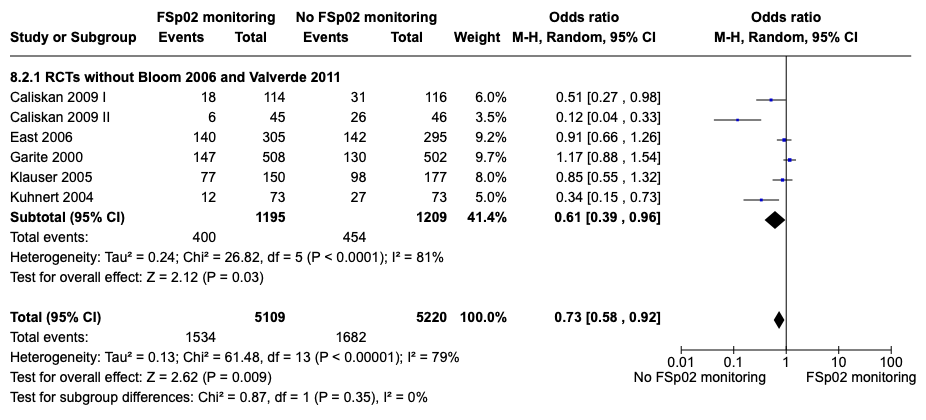
**

Figure 8. The association between addition of fetal oxygen saturation monitoring to fetal heart rate monitoring and delivery by Cesarean section. Subgroup Analysis.


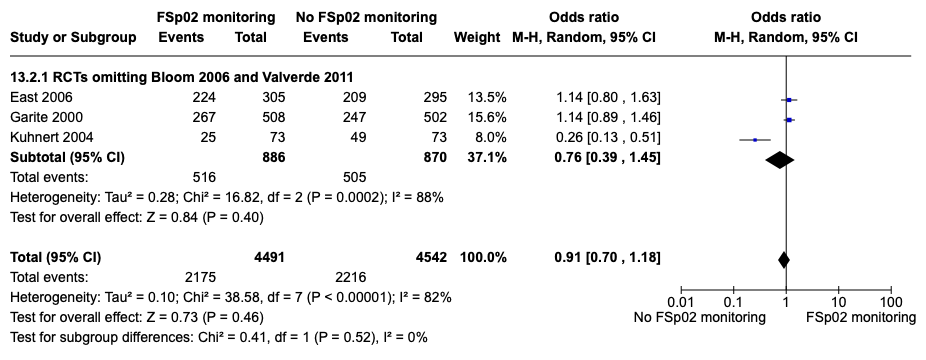


Figure 9. The association between addition of fetal oxygen saturation monitoring to fetal heart rate monitoring and operative deliveries (Cesarean sections and operative vaginal deliveries). Subgroup Analysis


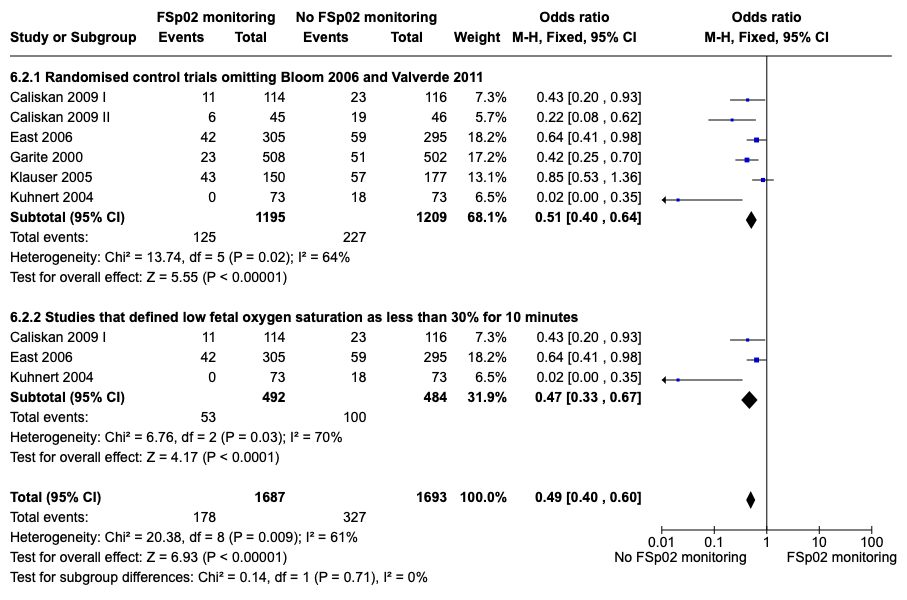


Figure 10. The association between the addition of fetal oxygen saturation monitoring to fetal heart rate monitoring and Cesarean sections for non-reassuring fetal status. Subgroup analysis.


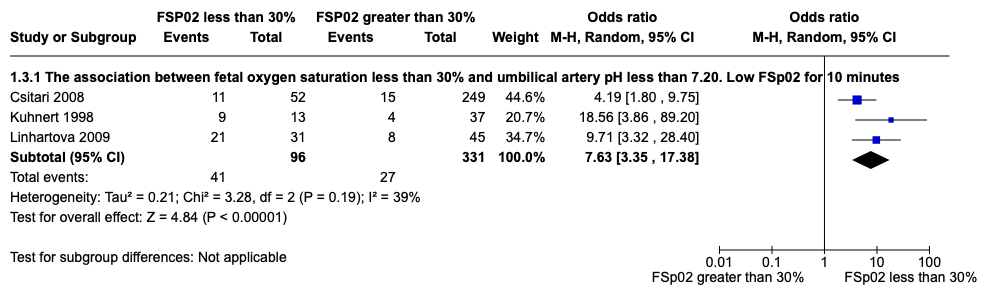


Figure 11. The association between fetal oxygen saturation less than 30% for 10 minutes and umbilical artery pH less than 7.20


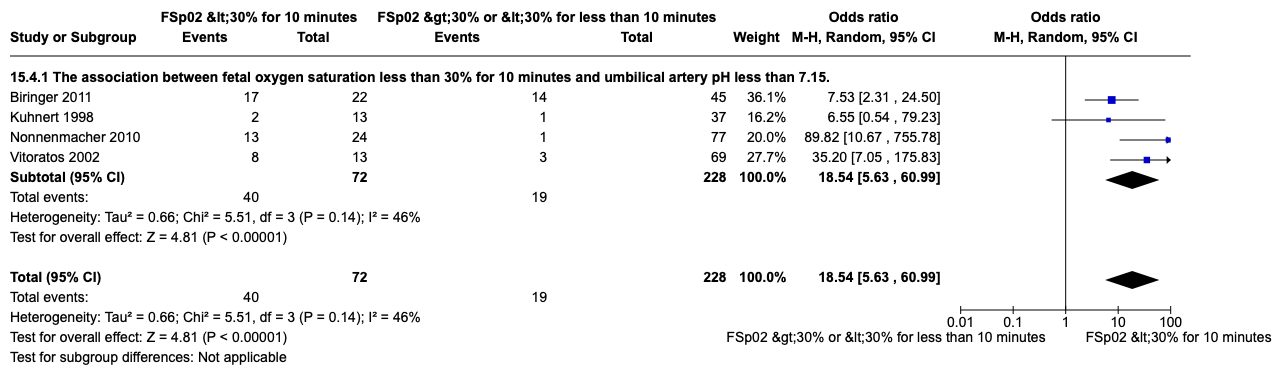


Figure 12. The association between fetal oxygen saturation less than 30% for 10 minutes and umbilical artery pH less than 7.15


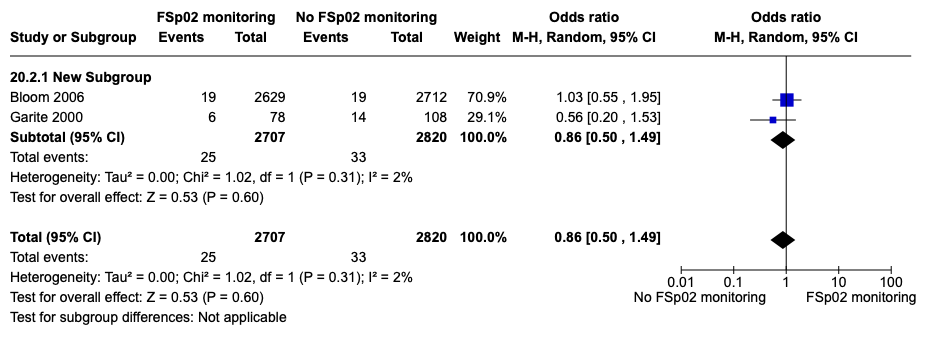


Figure 13. Neonatal intubation: use of fetal pulse oximetry and fetal heart rate monitoring versus fetal heart rate monitoring without fetal pulse oximetry. Term Infants Only


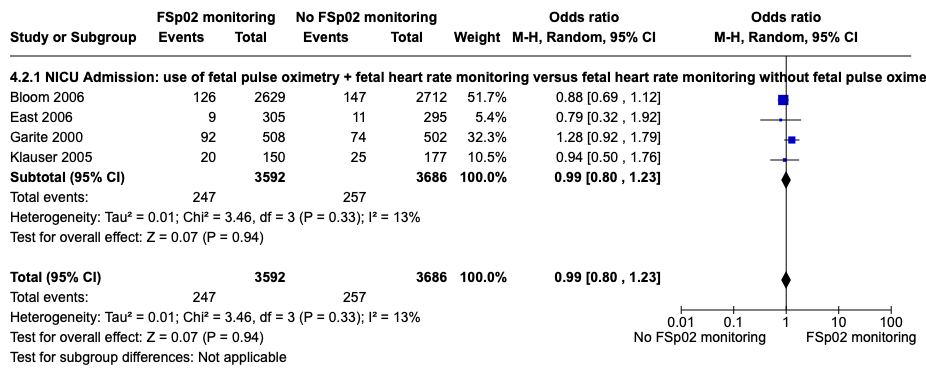


Figure 14. NICU Admission: use of fetal pulse oximetry and fetal heart rate monitoring versus fetal heart rate monitoring without fetal pulse oximetry. Term Infants Only


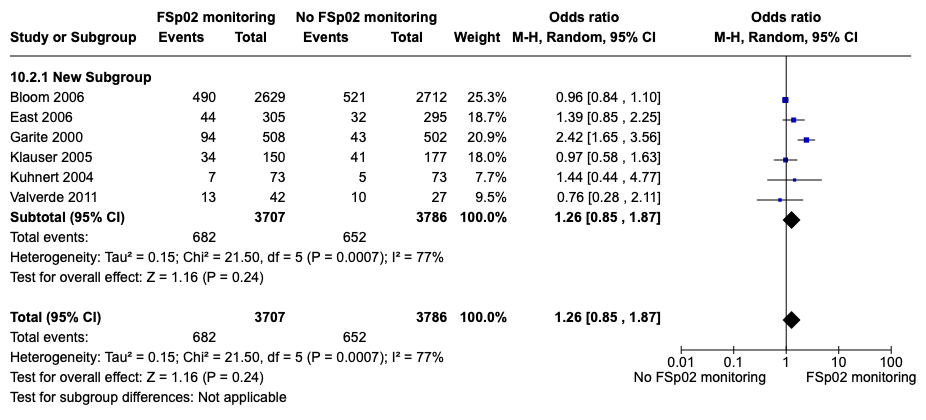


 Figure 15. Cesarean section for dystocia in labour: use of fetal pulse oximetry versus not using fetal pulse oximetry. Term Infants only.


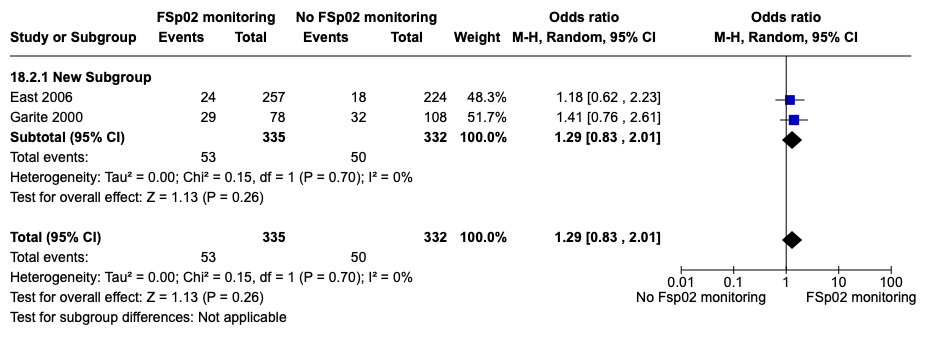


Figure 16. The association between addition of FSp02 monitoring to FHR monitoring and umbilical artery base excess of less than -10mmol/L. Term infants only.


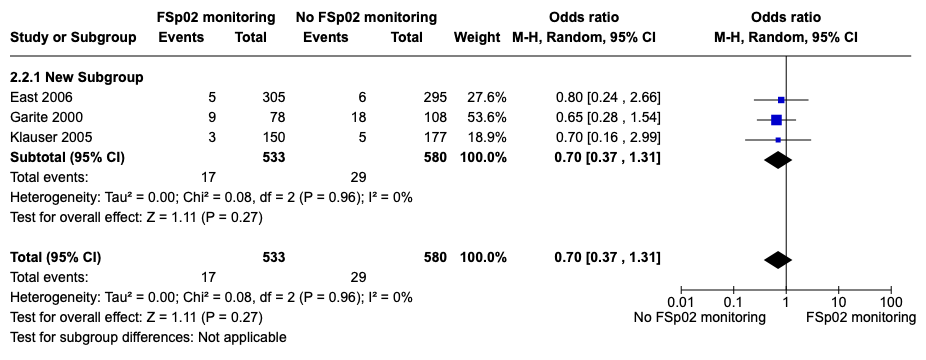


Figure 17. Association between addition of FSp02 monitoring to FHR monitoring and 5 minute Apgar scores of less than 7. Term infants only.


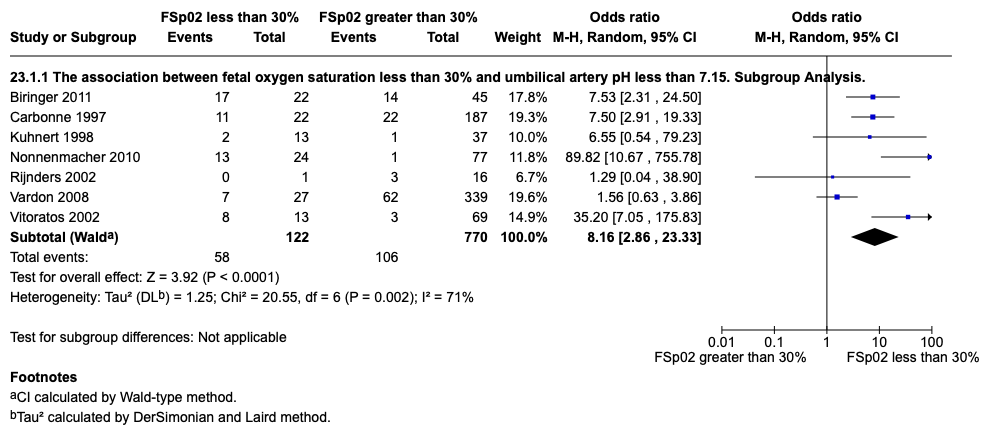


Figure 19. The association between fetal oxygen saturation less than 30% and umbilical artery pH less than 7.15. Subgroup analysis excluding studies that defined abnormal FSp02 as ≤30%


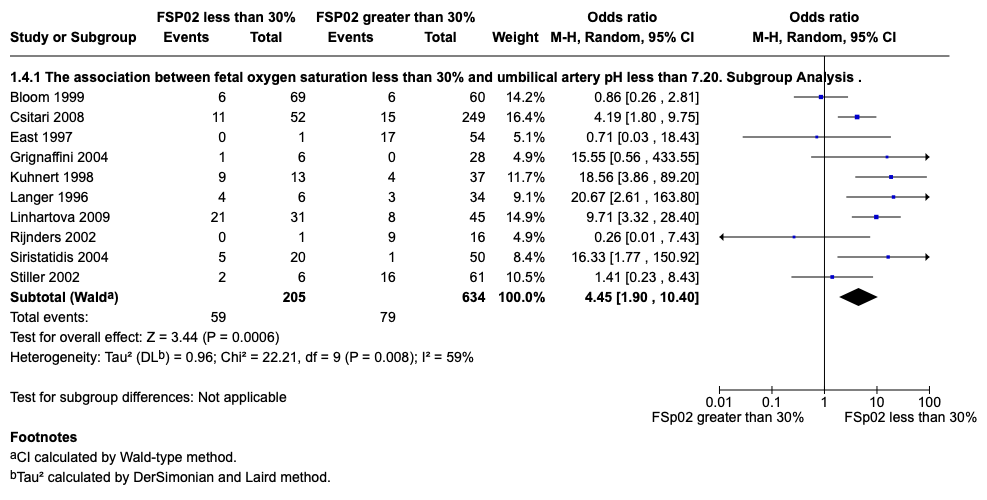


Figure 19. The association between fetal oxygen saturation less than 30% and umbilical artery pH less than 7.20. Subgroup analysis excluding studies that defined abnormal FSp02 as ≤30%


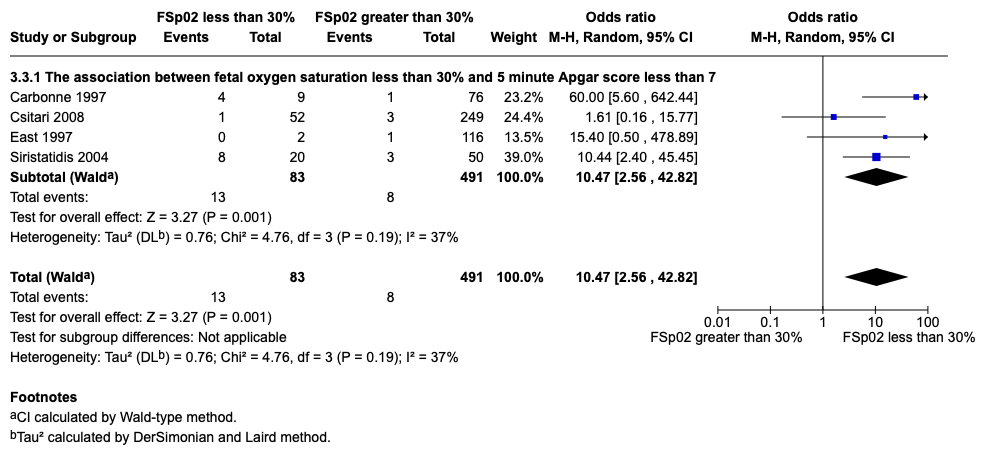


Figure 20. The association between fetal oxygen saturation less than 30% and 5 minute Apgar score <7. Subgroup analysis excluding studies that defined abnormal FSp02 as ≤30%


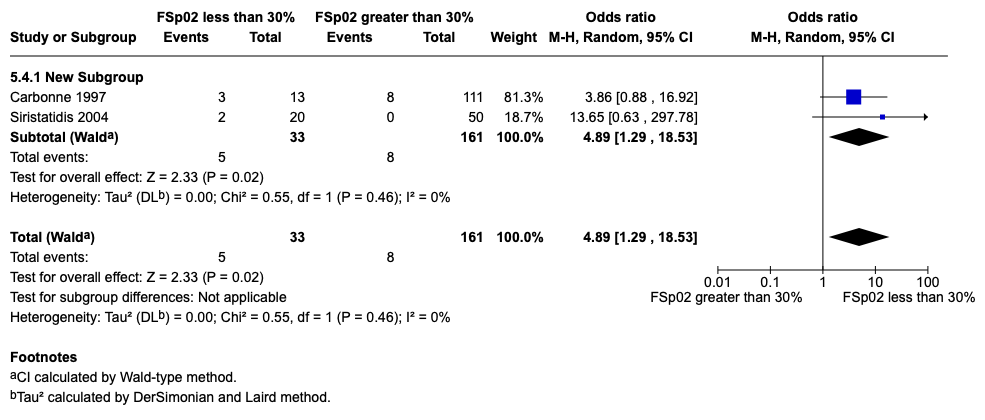


Figure 21. The association between fetal oxygen saturation less than 30% and admission to the neonatal intensive care unit. Subgroup analysis excluding studies that defined abnormal FSp02 as ≤30%

# Appendix 10. Prediction Intervals

Table 1. The association between fetal oxygen saturation less than 30% and adverse neonatal outcomes. Prediction Intervals.

| **Outcome** | **Mean** | **Lower limit of prediction interval** | **Higher limit of prediction interval** |
| --- | --- | --- | --- |
| **Umbilical artery pH <7.15** | 7.86 | 0.56 | 110.18 |
| **Umbilical artery pH <7.15. Cohort studies only** | 6.59 | 0.37 | 118.99 |
| **Umbilical artery pH <7.15. Good quality studies only** | 8.17 | 0.12 | 577.10 |
| **Umbilical artery pH <7.20** | 5.53 | 0.312 | 98.18 |
| **Umbilical artery pH <7.20. Good quality studies only** | 8.17 | 0.12 | 577.10 |
| **5-minute Apgar score <7** | 16.63 | 1.32 | 209.95 |
| **5-minute Apgar score <7. Good quality studies only** | 24.45 | 2.92 | 205.00 |
| **NICU admission** | 5.89 | 0.01 | 16342.33 |

Table 2. The association between the addition of FSp02 monitoring to standard monitoring and adverse neonatal and delivery outcomes. Prediction Intervals.

| **Outcome** | **Mean** | **Lower limit of prediction interval** | **Higher limit of prediction interval** |
| --- | --- | --- | --- |
| **Umbilical artery pH <7.20** | 0.88 | 0.01 | 11340.0 |
| **Umbilical artery pH <7.15** | 0.90 | 0.01 | 111.25 |
| **Umbilical artery pH <7.0** | 0.91 | 0.22 | 3.73 |
| **Base Excess <10mmol/L** | 1.26 | 0.07 | 21.38 |
| **5-minute Apgar <7** | 0.66 | 0.26 | 1.67 |
| **5-minute Apgar <7. Term infants only** | 0.70 | 0.01 | 40.69 |
| **NICU admission** | 0.98 | 0.29 | 3.27 |
| **NICU admission. Term infants only** | 0.99 | 0.52 | 1.88 |
| **Neonatal intubation** | 0.89 | 0.04 | 22.03 |
| **Operative delivery (RCTs)** | 0.96 | 0.31 | 2.99 |
| **Cesarean section** | 0.79 | 0.30 | 2.06 |
| **Cesarean section for non-reassuring fetal status** | 0.59 | 0.19 | 1.80 |
| **Cesarean section for non-reassuring fetal status. Subgroup analysis excluding Bloom 2006 and Valverde 2011** | 0.51 | 0.37 | 0.70 |
| **Cesarean section for dystocia** | 1.07 | 0.93 | 1.23 |
| **Cesarean section for dystocia. Term infants only** | 1.26 | 0.38 | 4.23 |
| **Operative vaginal delivery for dystocia** | 1.97 | 0.07 | 54.75 |

# References

1. Bloom SL, Spong CY, Thom E, et al. Fetal pulse oximetry and cesarean delivery. New England journal of medicine 2006;355:2195‐202.

2. Caliskan E, Cakiroglu Y, Corakci A, Ozeren S. Reduction in Cesarean delivery with fetal heart rate monitoring and intermittent pulse oximetry after induction of labour with misoprostol. Journal of Maternal-Fetal and Neonatal Medicine 2009;22:445-51.

3. Çalişkan E, Doǧer E, Çakiroǧlu Y, Çorakçi A, Özeren S. The effect of fetal pulse oximetry on neonatal outcomes of fetuses with the diagnosis of intrauterine growth restriction. Turk Jinekoloji ve Obstetrik Dernegi Dergisi 2009;6:35-40.

4. East CE, Brennecke SP, King JF, Chan FY, Colditz PB. The effect of intrapartum fetal pulse oximetry, in the presence of a nonreassuring fetal heart rate pattern, on operative delivery rates: A multicenter, randomized, controlled trial (the FOREMOST trial). American Journal of Obstetrics and Gynecology 2006;194:606.e1-06.e16.

5. Garite TJ, Dildy GA, McNamara H, Nageotte MP, Boehm FH, Dellinger EH. A multicenter controlled trial of fetal pulse oximetry in the intrapartum treatment of nonreassuring fetal heart rate patterns. American journal of obstetrics and gynecology 2000;183:1049‐58.

6. Klauser CK, Christensen EE, Chauhan SP, Bufkin L, Magann EF, Bofill JA. Use of fetal pulse oximetry among high-risk women in labour: a randomized clinical trial. American journal of obstetrics and gynecology 2005;192:1810‐19.

7. Kühnert M, Schmidt S. Intrapartum management of nonreassuring fetal heart rate patterns: A randomized controlled trial of fetal pulse oximetry. American Journal of Obstetrics and Gynecology 2004;191:1989-95.

8. Valverde M, Puertas AM, Lopez-Gallego MF, Carrillo MP, Aguilar MT, Montoya F. Effectiveness of pulse oximetry versus fetal electrocardiography for the intrapartum evaluation of nonreassuring fetal heart rate. European Journal of Obstetrics and Gynecology and Reproductive Biology 2011;159:333-37.

9. Bakr AF, Al-Abd M, Karkour T. Fetal pulse oximetry and neonatal outcome: A study in a developing country. Journal of Perinatology 2005;25:759-62.

10. Biringer K, Danko J, Žúbor P, Matašová K, Zibolen M, Pullmann R. Biophysical methods in diagnosis of intrapartal fetal hypoxia. Ceska Gynekologie 2011;76:222-29.

11. Bloom SL, Swindle RG, McIntire DD, Leveno KJ. Fetal pulse oximetry: Duration of desaturation and intrapartum outcome. Obstetrics and Gynecology 1999;93:1036-40.

12. Butterwegge M. Fetal pulse oximetry and non-reassuring heart rate. European Journal of Obstetrics and Gynecology and Reproductive Biology 1997;72:S63-S66.

13. Carbonne B, Audibert F, Segard L, Sebban E, Cabrol D, Papiernik E. Fetal pulse oximetry: Correlation between changes in oxygen saturation and neonatal outcome. Preliminary report on 39 cases. European Journal of Obstetrics and Gynecology and Reproductive Biology 1994;57:73-77.

14. Carbonne B, Langer B, Goffinet F, et al. Multicenter study on the clinical value of fetal pulse oximetry: II. Compared predictive values of pulse oximetry and fetal blood analysis. American Journal of Obstetrics and Gynecology 1997;177:593-98.

15. Chua S, Yam J, Razvi K, Yeong SM, Arulkumaran S. Intrapartum fetal oxygen saturation monitoring in a busy labour ward. European Journal of Obstetrics and Gynecology and Reproductive Biology 1999;82:185-89.

16. Csitári IK, Pasztuhov A, László A. The reliability of fetal pulse oximetry: The effect of fetal oxygen saturation below 30% on perinatal outcome. European Journal of Obstetrics and Gynecology and Reproductive Biology 2008;136:160-64.

17. East CE, Dunster KR, Colditz PB, Nath CE, Earl JW. Fetal oxygen saturation monitoring in labour: An analysis of 118 cases. Australian and New Zealand Journal of Obstetrics and Gynaecology 1997;37:397-401.

18. Fernández Andrés I, Martínez Montero I. Fetal pulse oximetry. Intrapartum foetal hypoxia evaluation. Comparative study with invasive techniques concerning foetal welfare. Anales del sistema sanitario de Navarra 2004;27:179‐89.

19. Grignaffini A, Soncini E, Ronzoni E, Piazza E, Anfuso S, Vadora E. Meconium-stained amniotic fluid and fetal oxygen saturation measured by pulse oximetry during labour. Acta Biomedica de l'Ateneo Parmense 2004;75:45-52.

20. Hájek Z, Srp B, Pavlíková M, et al. Intrapartal fetal monitoring, sensitivity and specificity of methods. Ceska Gynekologie 2006;71:263-67.

21. Kuhnert M, Seelbach-Gobel B, Butterwegge M. Predictive agreement between the fetal arterial oxygen saturation and fetal scalp pH: results of the German multicenter study. Am J Obstet Gynecol 1998;178:330-5.

22. Kühnert M, Seelbach-Göbel B, Butterwegge M. Fetal pulse oximetry in clinical use. Geburtshilfe und Frauenheilkunde 2001;61:290-96.

23. Langer B, Boudier E, Haddad J, Pain L, Schlaeder G. Fetal pulse oximetry during labor of 62 patients. Fetal Diagnosis and Therapy 1996;11:37-45.

24. Linhartova L, Kurtansky A, Suska P. Correlation between fetal blood oxygen saturation and umbilical blood pH values. Bratislava Medical Journal 2009;110:684-87.

25. Luttkus AK, Dudenhausen JW. Fetal pulse oximetry. Bailliere's Clinical Obstetrics and Gynaecology 1996;10:295-306.

26. Luttkus AK, Lübke M, Büscher U, Porath M, Dudenhausen JW. Accuracy of fetal pulse oximetry. Acta Obstet Gynecol Scand 2002;81:417-23.

27. Luttkus AK, Stupin JH, Callsen TA, Dudenhausen JW. Feasibility of simultaneous application of fetal electrocardiography and fetal pulse oximetry. Acta Obstetricia et Gynecologica Scandinavica 2003;82:443-48.

28. McNamara H, Chung DC, Lilford R, Johnson N. Do fetal pulse oximetry readings at delivery correlate with cord blood oxygenation and acidaemia? British Journal of Obstetrics and Gynaecology 1992;99:735-38.

29. Nikolov A, Dimitrov A, Iarukova N, Vakrilova L, Krusteva K. Intrapartum oxygen saturation in fetus with symptoms of distress shown during fetal cardiotocograph monitoring. Akusherstvo i ginekologiia 2004;43:3-10.

30. Nonnenmacher A, Hopp H, Dudenhausen J. Predictive value of pulse oximetry for the development of fetal acidosis. Journal of Perinatal Medicine 2010;38:83-86.

31. Rijnders RJ, Mol BW, Reuwer PJ, Drogtrop AP, Vernooij MM, Visser GH. Is the correlation between fetal oxygen saturation and blood pH sufficient for the use of fetal pulse oximetry? J Matern Fetal Neonatal Med 2002;11:80-3.

32. Roztocil A, Miklica J, Kucera M, Ventruba P. Continuous monitoring of fetal oxygen saturation (FSpO2) using intrapartum fetal pulse oximetry (IFPO) in the diagnosis of acute fetal hypoxia. Ceská gynekologie / Ceská lékarská spolecnost J Ev Purkyne 2000;65:224-30.

33. Salamalekis E, Vitoratos N, Loghis C, Panayotopoulos N, Kassanos D, Creatsas G. Evaluation of fetal heart rate patterns during the second stage of labor through fetal oximetry. Gynecologic and Obstetric Investigation 1999;48:151-54.

34. Seelbach-Gobel B, Butterwegge M, Kuhnert M, Heupel M. Fetal reflectance pulse oximetry during labour. Zeitschrift fur Geburtshilfe und Perinatologie 1994;198:67-71.

35. Seelbach-Gobel B, Heupel M, Kuhnert M, Butterwegge M. The prediction of fetal acidosis by means of intrapartum fetal pulse oximetry. American Journal of Obstetrics and Gynecology 1999;180:73-81.

36. Seelbach-Göbel B, Riedl T. [Reliability of fetal pulse oximetry for the detection of fetal acidosis]. Z Geburtshilfe Neonatol 2005;209:43-50.

37. Siristatidis C, Salamalekis E, Vitoratos N, et al. Intrapartum surveillance of IUGR fetuses with cardiotocography and fetal pulse oximetry. Biology of the Neonate 2003;83:162-65.

38. Siristatidis C, Salamalekis E, Kassanos D, Loghis C, Creatsas G. Evaluation of fetal intrapartum hypoxia by middle cerebral and umbilical artery Doppler velocimetry with simultaneous cardiotocography and pulse oximetry. Archives of gynecology and obstetrics 2004;270:265-70.

39. Skoczylas M, Laudański T. Usefulness of the examination of fetal blood oxygen saturation (FSpO2) and fetal heart rate (FHR) as a prognostic factor of the newborn outcome. Ginekologia polska 2003;74:1284-89.

40. Sobotková D, Kučerová I, Dittrichová J, Velebil P. Psychomotor development of children with signs of intrapartum hypoxia and monitored by intrapartum fetal pulse oxymetry. Ceska Gynekologie 2004;69:114-20.

41. Sobotková D, Kucerová I, Dittrichová J, Velebil P. [Psychomotor development of children with signs of intrapartum hypoxia and monitored by intrapartum fetal pulse oxymetry]. Ceska Gynekol 2004;69 Suppl 1:114-20.

42. Stiller R, Mering RV, König V, Huch A, Huch R. How well does reflectance pulse oximetry reflect intrapartum fetal acidosis? American Journal of Obstetrics and Gynecology 2002;186:1351-57.

43. Tomialowicz M, Zimmer M, Pomorski M, Fuchs T. Biophysical and biochemical assessment of fetal perinatal hypoxia. Advances in Clinical and Experimental Medicine 2007;16:249-55.

44. Uchida T, Kanayama N, Mukai M, et al. Examiner's finger-mounted fetal tissue oximetry: A preliminary report on 30 cases. Journal of Perinatal Medicine 2015;2015.

45. Vardon D, Hors Y, Grossetti E, Creveuil C, Herlicoviez M, Dreyfus M. Fetal pulse oximetry: Clinical practice. Journal de Gynecologie Obstetrique et Biologie de la Reproduction 2008;37:697-704.

46. Vitoratos N, Salamalekis E, Saloum J, Makrakis E, Creatsas G. Abnormal fetal heart rate patterns during the active phase of labor: The value of fetal oxygen saturation. Journal of Maternal-Fetal Medicine 2002;11:46-49.

47. Markwitz W, Ropacka M, Breborowicz GH. Fetal pulse oximetry in second stage of labor. Ginekologia polska 2000;71:218-23.

48. Leszczynska-Gorzelak B, Poniedzialek-Czajkowska E, Oleszczuk J. Fetal blood saturation during the 1st and 2nd stage of labor and its relation to the neonatal outcome. Gynecologic and Obstetric Investigation 2002;54:159-63.

49. East CE, Brennecke SP, Chan FY, King JF, Beller EM, Colditz PB. Clinicians' evaluations of fetal oximetry sensor placement in a multicentre randomised trial (the FOREMOST trial). Australian and New Zealand Journal of Obstetrics and Gynaecology 2006;46:234-39.

50. Butterwegge M. Fetal pulse oximetry and non-reassuring heart rate. European Journal of Obstetrics Gynecology and Reproductive Biology 1997;72:S63-S66.

51. Uchida T, Kanayama N, Mukai M, et al. Examiner's finger-mounted fetal tissue oximetry: a preliminary report on 30 cases. Journal of Perinatal Medicine 2016;44:745-49.

52. Wells G, Shea B, O'Connell D, et al. The Newcastle–Ottawa Scale (NOS) for Assessing the Quality of Non-Randomized Studies in Meta-Analysis. ᅟOttawa Hospital Research Institute 2000;ᅟ.

53. Kuhnert M, Schmidt S. Intrapartum management of nonreassuring fetal heart rate patterns: A randomized controlled trial of fetal pulse oximetry. American Journal of Obstetrics and Gynecology 2004;191:1989-95.

54. Valverde Pareja M, Martínez MDM, Guadix BR, Ventoso FM, Prieto AP, Criado MSL. Comparison between the pulse oximetry's effectiveness and electrocardiogram's effectiveness ; Two methods of fetal intrapartum monitoring. Journal of Maternal-Fetal and Neonatal Medicine 2010;23:627.

55. Valverde M, Puertas AM, Lopez-Gallego MF, Carrillo MP, Aguilar MT, Montoya F. Effectiveness of pulse oximetry versus fetal electrocardiography for the intrapartum evaluation of nonreassuring fetal heart rate. European journal of obstetrics, gynecology, and reproductive biology 2011;159:333‐37.
